# Action Interpretation Determines the Effects of Go/No-Go and Approach/Avoidance Actions on Stimulus Evaluation

**DOI:** 10.1162/opmi_a_00151

**Published:** 2024-07-19

**Authors:** Zhang Chen, Pieter Van Dessel

**Affiliations:** Department of Experimental Psychology, Ghent University, Ghent, Belgium; Department of Experimental Clinical and Health Psychology, Ghent University, Ghent, Belgium

**Keywords:** go/no-go, approach/avoidance, evaluation, action execution, action interpretation

## Abstract

Executing go/no-go or approach/avoidance responses toward a stimulus can change its evaluation. To explain these effects, some theoretical accounts propose that executing these responses inherently triggers affective reactions (i.e., action execution), while others posit that the evaluative influences originate from interpreting these responses as valenced actions (i.e., action interpretation). To test the role of action execution and action interpretation in these evaluative effects, we developed a novel training task that combined both go/no-go and approach/avoidance actions orthogonally. Participants either responded or did not respond (i.e., go/no-go) to control a shopping cart on screen, and as a result, either collected or did not collect (i.e., approach/avoidance) certain food items. When the task instructions referred to the go/no-go actions (Experiment 1, *N* = 148), we observed an effect of these actions. Participants evaluated no-go items less positively than both go and untrained items. No effect of approach/avoidance actions was observed. Contrarily, when the task instructions referred to the approach/avoidance actions (Experiment 2, *N* = 158), we observed an approach/avoidance effect. Participants evaluated approached items more positively and avoided items less positively than untrained items. No effect of go/no-go actions was observed. This suggests that action interpretation determined whether go/no-go or approach/avoidance actions influenced stimulus evaluation, when the same motor responses were made. Further examination of the role of action interpretation can inform theories of how actions influence stimulus evaluation, and facilitate the use of these interventions in applied settings.

## INTRODUCTION

Our likes and dislikes for an object can influence how we react to it. Interestingly, the opposite direction also seems to hold: our responses toward an object may influence how much we like or dislike it afterwards. This latter observation has inspired several (computer-based) behavioral interventions to change people’s evaluation of certain objects (e.g., addictive or harmful stimuli), by training them to execute certain responses toward these objects.

Two such training tasks are the go/no-go training (GNG) and the approach/avoidance training (AAT). In the GNG, some stimuli are consistently paired with a go cue, so that people repeatedly respond to them, by for instance pressing a key on a keyboard. Other stimuli are consistently paired with a no-go cue, so that people do not respond to them (Veling, Lawrence, et al., 2017). In the AAT, some stimuli are consistently paired with an approach cue and others with an avoidance cue. As a result, people are trained to repeatedly approach or avoid a certain stimulus. Different variants of the AAT exist. For instance, in the joystick version of the task, participants approach a stimulus by pulling a joystick toward themselves (often accompanied by the perceptual feedback of the stimulus becoming larger on screen), and avoid a stimulus by pushing the joystick away from themselves (often accompanied by the stimulus becoming smaller; e.g., Becker et al., 2015; Veling et al., 2021). In the manikin version of the task, participants press keys on a keyboard to move a manikin on screen either closer to (i.e., approach) or further away (i.e., avoidance) from a stimulus (e.g., Van Dessel, Hughes, & De Houwer, 2018; Woud et al., 2013).

Both the GNG and AAT have been shown to change people’s evaluation of trained stimuli. After the GNG, participants typically evaluate no-go stimuli less positively than both go stimuli and stimuli that are not included in the training (untrained items; e.g., Chen et al., 2016, 2018; for a meta-analysis on the effect of GNG on food evaluation, see Yang et al., 2022). We will term this effect the no-go devaluation effect, and define it as the more negative evaluation of no-go items compared to both go and untrained items (see e.g., Chen et al., 2016). After the AAT, avoided stimuli are generally evaluated less positively than approached stimuli (Van Dessel, De Houwer, & Gast, 2016; Van Dessel, Eder, & Hughes, 2018). These observations raised the question of how performing go/no-go or approach/avoidance actions may impact stimulus evaluation, a question that we turn to next.

### How Do Go/No-Go and Approach/Avoidance Actions Change Stimulus Evaluation?

The GNG and AAT share some procedural similarities but also differences. Both tasks train people to repeatedly execute a certain (non-)response toward a stimulus, but the nature of the trained (non-)responses differs. Most previous work has examined the two tasks separately. Consequently, different theoretical accounts have been developed for how go/no-go and approach/avoidance actions may influence stimulus evaluation.

For the GNG, the Behavior Stimulus Interaction (BSI) theory proposes that an appetitive stimulus triggers a strong tendency to respond. However, when the stimulus is paired with a no-go cue, this tendency to respond needs to be inhibited to fulfil the task requirement (Veling et al., 2008, 2022). The resulting response conflict then triggers negative affective reactions (Dignath et al., 2020; Dreisbach & Fischer, 2012). Repeatedly experiencing negative affect when encountering an initially appetitive no-go stimulus thus makes it less attractive afterwards. While the BSI theory ascribes the devaluation of no-go items to response conflict on no-go trials, no-go actions have also been posited to be inherently negative, due to the hardwired connection between no-go responses and punishment (Guitart-Masip et al., 2014), or between ‘stopping’ and the Pavlovian aversive system (Verbruggen et al., 2014). Executing no-go responses thus triggers aversive affective reactions, which are then attached to a no-go stimulus and reduce its subsequent evaluation. We will term this account the ‘devaluation-by-no-go’ account here. Note that making speeded go responses (e.g., by implementing a strict response deadline) has been shown to increase the evaluation of (Chen et al., 2016) and preference for go stimuli (Schonberg et al., 2014; Veling, Chen, et al., 2017). This go valuation effect is defined as a more positive evaluation of go items compared to both no-go and untrained items. The go valuation effect may be explained by an inherent link between (invigorated) go responses and reward (Guitart-Masip et al., 2014; Verbruggen et al., 2014), and/or increased attention toward go items when responding rapidly (Itzkovitch et al., 2022; Schonberg & Katz, 2020). However, since most studies using the GNG did not implement a strict response deadline on go trials and did not observe a go valuation effect, we will not further discuss the go valuation effect here, but will come back to this phenomenon in the General Discussion.

Similarly, for the AAT, different accounts have been proposed to explain its effect on stimulus evaluation. According to the motivational systems account (Neumann et al., 2003), positively valenced stimuli are linked to an approach motivational orientation, and negatively valenced stimuli are linked to an avoidance motivational orientation. The links between positive/negative valence and approach/avoidance motivational orientation are assumed to be bidirectional. Thus, approaching and avoiding a stimulus can trigger positive and negative affective reactions respectively, which then change the evaluation of the stimulus. The common coding account (Eder & Klauer, 2009; Eder & Rothermund, 2008) instead proposes that task instructions and task goals (such as the response labels of *toward* and *away*, as typically used to describe approach and avoidance actions) imbue certain motor responses with evaluative connotations. These valence codes then become part of the representation of these motor responses, and become activated when the motor responses are executed. The evaluation of a stimulus is changed by repeatedly co-activating a positive or negative valence code. Lastly, the inferential account (Van Dessel et al., 2019) proposes that during the AAT, participants may form propositions such as ‘*I am approaching stimulus A and avoiding stimulus B*’. This proposition, combined with the proposition that ‘*Pleasant stimuli are generally approached and unpleasant stimuli are generally avoided*’ may allow them to infer (i.e., inferential reasoning) that they like (or are assumed to like) the approached stimulus and dislike the avoided stimulus, thereby changing their evaluation of these stimuli.

### The Evaluative Origins of Go/No-Go and Approach/Avoidance Actions: Action Execution or Action Interpretation?

The brief overview above reveals some conceptual similarities but also differences among the current theoretical accounts on how the GNG and AAT influence stimulus evaluation. One dimension on which these accounts differ, also the one that we will focus on here, is the origin of the evaluative influences of go/no-go and approach/avoidance actions. Both the BSI theory and the devaluation-by-no-go account for the GNG, and the motivational systems account for the AAT, emphasize the nature of executed motor responses (i.e., action execution). According to these accounts, making no-go or avoidance responses may inherently trigger negative affective reactions, whereas making approach responses (and under some circumstances, making go responses; see above) may inherently trigger positive affective reactions. In contrast, the common coding account and the inferential account for the AAT ascribe the evaluative origins of approach and avoidance responses to how people interpret these actions. According to these latter accounts, the same motor responses can be interpreted differently depending on the context, such as what labels people use to describe these responses. Crucially, action interpretation, rather than action execution per se, determines their evaluative influences, by changing either the valence code in action representation (Eder & Klauer, 2009; Eder & Rothermund, 2008), or guiding the inferences that people make (Van Dessel et al., 2019).

Previous work has shown that action interpretation manipulated via response labels influences the AAT effect (Van Dessel, Hughes, & De Houwer, 2018). In the manikin AAT, participants liked approached stimuli more than avoided stimuli when their responses were labeled as approach (i.e., distance decrease between the manikin and a stimulus) and avoidance (i.e., distance increase between the manikin and a stimulus). However, when the same actions were labeled as moving the manikin either *upward* or *downward* on screen, participants liked upward stimuli more than downward stimuli, which may be explained by the positive connotation of *upward* and the negative connotation of *downward* (Eder & Rothermund, 2008). Importantly, when the responses were framed as upward/downward movements but not approach/avoidance actions, changes in distance did not influence stimulus evaluation. Action interpretation, rather than features of action execution, thus seemed to determine the AAT effect.

Juxtaposing the theoretical accounts for the AAT and GNG effects may leave the impression that the evaluative impact of approach and avoidance actions originates from action interpretation (Van Dessel, Hughes, & De Houwer, 2018), whereas that of go and no-go actions originates from action execution. After all, the two current theoretical accounts for the no-go devaluation effect both emphasize the execution of no-go responses, rather than how the responses are framed and interpreted. However, this theoretical disparity may not necessarily mean that the evaluative origins of go/no-go and approach/avoidance actions differ. Instead, this may be a result of the two separate strands of research being developed mostly in isolation and having converged into different theoretical ideas. Recent work has started to compare the GNG and AAT both empirically (van Alebeek et al., 2023; Veling et al., 2021) and theoretically (Houben & Aulbach, 2023). One paper of special interest is that by Houben (2023), in which the labels for go and no-go responses were manipulated. One group of participants was instructed that making go responses meant to ‘take’ a food item and making no-go responses meant to ‘not take’ a food item (linking go to positive and no-go to negative valence), whereas another group was instructed that making go responses meant to ‘throw away’ a food item and making no-go responses meant to ‘keep’ a food item (linking go to negative and no-go to positive valence). The results showed an effect of response labels on the evaluation of chocolate stimuli but not fruit stimuli (i.e., the two types of food items used in the training). Chocolate stimuli were liked less when paired with a negative response label compared to a positive response label. Furthermore, no effect of go and no-go responses on evaluation was observed (Houben, 2023). This finding suggested that just like the AAT, the GNG effect on stimulus evaluation could be modulated by how go and no-go actions were interpreted. Most studies employing the GNG did not use the response labels used in Houben (2023). However, it is possible that participants might naturally interpret go as ‘take’ and no-go as ‘do not take’. Furthermore, words indicating action are generally perceived to be more positive than words indicating inaction (McCulloch et al., 2012). Merely labeling responses as ‘go’ or ‘no-go’ might therefore be sufficient to imbue them with evaluative meaning.

The findings by Houben (2023) provided some initial evidence for a role of action interpretation in the GNG effect. However, a potential effect of go and no-go action execution could not be fully ruled out, even though no overall effect of go versus no-go responses was observed. This is because the response labels used in the two groups, namely ‘take’/‘do not take’ and ‘throw away’/‘keep’, may not be exactly matched in their evaluative meanings, which might disguise a potential effect of go and no-go responses. For instance, throwing something away might carry more negative connotation than simply not taking it, which might lead to the prediction that ‘throw away’ items should be evaluated less positively than ‘do not take’ items. However, descriptively, the opposite was observed in Houben (2023): no-go-‘do not take’ items were evaluated less positively than go-‘throw away’ items. This observation suggested that an effect of go/no-go responses might still exist, and might to some extent have overcome the evaluative difference between ‘throw away’ and ‘do not take’ labels. The potential roles of action execution and interpretation in the GNG effect thus warrant further research.

### The Current Research

The existing literature appeared to provide more evidence for the role of action interpretation in the AAT effect (e.g., Eder & Klauer, 2009; Eder & Rothermund, 2008; Van Dessel, Eder, & Hughes, 2018; Van Dessel et al., 2019) than in the GNG effect (Houben, 2023). It is unclear though whether the evaluative origins of go/no-go and approach/avoidance actions indeed differ. Studies on the GNG and AAT often differed on several methodological aspects (Veling et al., 2021), which made the direct comparison between the two difficult. Directly comparing the two training tasks on the evaluative origins of the trained actions can be very useful though, for both theoretical and practical reasons. From a theoretical perspective, identifying potential commonalities can allow researchers to develop more general theories of how simple motor responses influence stimulus evaluation, whereas finding possible differences can in turn lead to novel questions on how such differences may arise. These theoretical insights will also inform the use of the training tasks in more applied settings. Both the GNG and AAT have been used as behavior change interventions in various health domains, with outcome measures that often went beyond stimulus evaluation. For instance, previous work has examined the effects of GNG on eating behavior (e.g., Aulbach et al., 2021; Lawrence et al., 2015; Veling et al., 2014), smoking behavior (e.g., Bos et al., 2019; Scholten et al., 2019), and alcohol consumption (e.g., Jones et al., 2018; Schenkel et al., 2023) etc. Similarly, the effects of AAT have been investigated in the domain of eating behavior (e.g., Becker et al., 2015; Veling et al., 2021), alcohol consumption (e.g., Wiers et al., 2010, 2011), and prejudice toward certain individuals or groups (Kawakami et al., 2007; Van Dessel et al., 2020). While the results of some of these studies showed promise (e.g., in clinical contexts; Wiers et al., 2023), the results obtained were not always consistent (e.g., Cristea et al., 2016). A better understanding of the evaluative origins of go/no-go and approach/avoidance actions can help researchers and practitioners optimize the training procedures, and potentially develop novel interventions that combine the strengths of both tasks. Such improvements may also help make training-induced behavior changes more long-lasting, which is a key challenge for the research field (Chen & Veling, 2022).

In the current research, we examined the role of action execution and action interpretation in the GNG and AAT effects. To do this, we developed a novel training task that combined go/no-go and approach/avoidance actions in an orthogonal manner. Participants either pressed a key or did not press any key to control the location of a virtual shopping cart on screen (i.e., go and no-go actions). As a consequence of their go or no-go responses, their shopping cart either approached or avoided the image of a food item (i.e., there was a distance decrease or increase), similar to how approach and avoidance actions are defined in the manikin AAT (see the Methods section below for more details). In Experiment 1, participants were informed that the cues indicated whether they needed to make go or no-go actions to certain items, whereas in Experiment 2, the cues indicated whether they needed to make approach or avoidance actions. The motor responses in the two versions of the task were the same, yet in Experiment 1 they were referred to as go or no-go actions, whereas in Experiment 2 they were referred to as approach or avoidance actions. Note that our experimental strategy differed from that of Houben (2023). Instead of using labels of reversed valence to change action interpretation, we used response cues to manipulate action interpretation along one of two orthogonal dimensions (go/no-go vs. approach/avoidance). If executing a certain action inherently carries affective meaning, we would expect to see an effect of a certain action dimension even when the cues discourage action interpretation along this dimension (e.g., a no-go devaluation effect even when the cues lead people to interpret the actions as approach and avoidance). However, if we observed no effect of a certain action dimension when the response cues did not refer to this dimension, this would argue against the theoretical accounts that emphasized action execution, at least in their current forms. Auxiliary processes may need to be entertained, to explain such a boundary condition within the existing theoretical frameworks.

## EXPERIMENT 1

In Experiment 1, we used cues that indicated to participants whether they should make go or no-go responses. We therefore expected that they would interpret their responses as go and no-go actions, and expected a no-go devaluation effect. For whether approach and avoidance actions (i.e., the orthogonal dimension that was not indicated by the cues) would impact evaluation, we did not have a directional hypothesis.

### Ethics, Transparency, and Openness

The current research was conducted according to the ethical rules presented in the General Ethical Protocol of the Faculty of Psychology and Educational Sciences of Ghent University. All participants agreed to an informed consent (by clicking on a button that says ‘I agree’) before starting the online experiments. The informed consent specified that participants needed to be at least 18 years old in order to participate.

We report how we determined our sample size, all data exclusions (if any), all manipulations, and all measures in the study (Simmons et al., 2012). All data, analysis code, research materials, and the Supplemental Materials are available at https://osf.io/ezgqw. The pre-registration of Experiment 1 is at https://osf.io/w25dx. The pre-registration of Experiment 2 is at https://osf.io/bwrsc.

### Methods

#### Sample Size.

We planned to recruit 160 participants. This sample size was determined by the number of credits that we had for this experiment (i.e., resource constraints; Lakens, 2022). Sensitivity analysis in G*Power (version 3.1.9.6; Faul et al., 2007) shows that with 160 participants, we have 80% power to detect effect sizes as small as Cohen’s *d*_*z*_ of 0.223 (with a two-sided paired-samples *t* test and an alpha level of .05). A recent meta-analysis has shown that the overall effect of GNG on explicit food evaluation is Hedges’ *g* = 0.285 (Yang et al., 2022). When the correlation between two repeated measures in a within-subject design is larger than 0.5, Cohen’s *d*_*z*_ will be larger than Cohen’s *d*_*av*_, of which Hedges’ *g* is the unbiased version (Lakens, 2013). Since the correlations between repeated measures in the current experimental context often exceed 0.5 (Yang et al., 2022), the effect of GNG on explicit stimulus evaluation in Cohen’s *d*_*z*_ will likely be larger than 0.285, which is larger than 0.233, the effect size from the sensitivity analysis. As such, this sample size offers sufficient statistical power for detecting the effect of go/no-go actions on stimulus evaluation, and leaves some room for potential exclusion.

#### Participants.

In total, 161 participants (136 females, 24 males, 1 non-binary; *M*_*age*_ = 18.81, *SD*_*age*_ = 1.78) took part in the experiment for course credit in March, 2023. Due to the experimenter’s error, we exceeded the planned sample size by one. No eligibility criteria were applied (except being at least 18 years old, as specified in the informed consent), as per regulation of the local institute, all students should be allowed to participate in experiments for course credits.

#### Apparatus and Materials.

The experiment was programmed in jsPsych (version 7.2.1; de Leeuw, 2015), and run in Google Chrome and Firefox. Other web browsers were disabled as they might lead to compatibility issues. Participants could do the experiment on a desktop or a laptop with a keyboard. To ensure that all elements in the experiment were visible, participants could only start the experiment if the height of their screen was at least 750 pixels, and the width was at least 1200 pixels. Ninety food images were selected from the Food-pics_Extended database (Blechert et al., 2019). These ninety images largely overlapped with the eighty images used in Chen et al. (2016), and depicted various food categories, such as full meals, sweets, fruits, vegetables etc. For the images used, see the OSF repository.

#### Procedure.

Participants signed up for the experiment in the local participation system. The experiment was administered online, and participants could initiate the experiment at any time and at any place. We instructed them to complete the experiment in a quiet environment without distractions. Participants needed to agree to an informed consent before they could start the experiment. They then typed in their age, gender^1^ and nationality.

##### Rating Before Training (∼5 Minutes).

Participants first received a rating task, in which 90 food images were presented one by one. For each image, they were asked how attractive the depicted food appeared to them at that very moment. They responded by moving a cursor along a 200-point slider (−100 = *Not at all*; 100 = *Very much*), and could advance to the next trial by clicking on a ‘continue’ button below the slider. The cursor always started in the middle of the slider (i.e., 0). Participants were required to click on the slider first before they could click on ‘continue’, to ensure that they could not skip through trials by repeatedly clicking on ‘continue’.

##### Stimulus Selection.

All 90 images were then ranked from the highest rating till the lowest. For each participant, the 40 food images with the highest ratings were selected. We selected the most attractive food images, to be in line with previous work on the GNG (e.g., Chen et al., 2016), and also because there is an interest in using GNG and AAT to reduce the evaluation of appetitive stimuli. The 40 selected images were divided into five sets, with the average ratings of these five sets matched as closely as possible. These five sets were then randomly assigned into five conditions, namely go-approach, go-avoidance, no-go-approach, no-go-avoidance and untrained.

##### Training: The ‘Food Shopping’ Game (∼30 Minutes).

**Figure 1.**
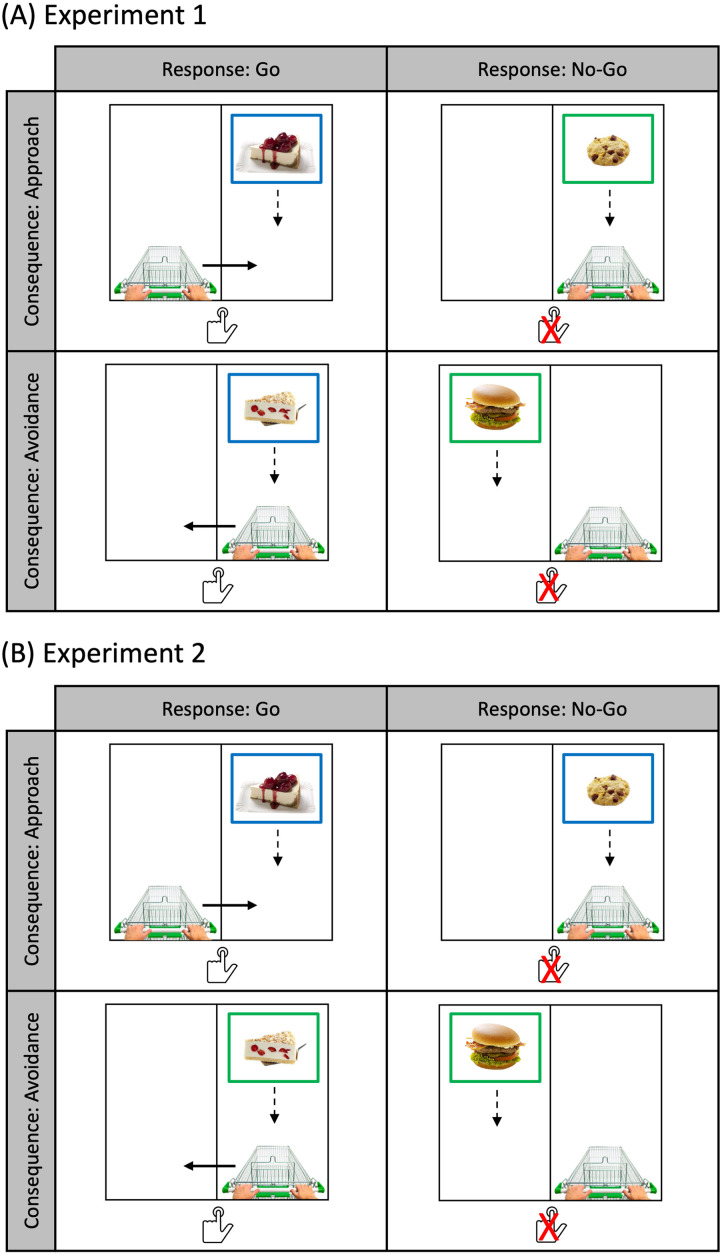
The training task in Experiment 1 (A) and 2 (B). The cues indicate go/no-go in Experiment 1, and approach/avoidance in Experiment 2. In this example, the blue cue indicates a go response (A) or an approach consequence (B), and the green cue indicates a no-go response (A) or an avoidance consequence (B). The dashed down arrows indicate how the images move from top to bottom. The solid arrows in the go-approach and go-avoidance conditions indicate how the cart moves after participants respond. The arrows are for illustration, and are not presented in the task. The food images are from the Food-Pics_Extended database (Blechert et al., 2019). The shopping cart image is from pngwing.com, and the hand pushing button image is from stockio.com.

Participants then received the training, called the ‘food shopping’ game. They were told that they were a customer in a virtual market. The market had two lanes, a left lane and a right lane. A shopping cart was presented at the bottom of one of the lanes, and participants were told that this shopping cart belonged to them (Figure 1). On each trial, a food image appeared near the top on either the left or the right lane, and gradually moved toward the bottom. The whole animation lasted 2 seconds. 300 milliseconds after image onset, a frame appeared around the image. The color of the frame determined whether participants should respond or not. We used two colors, green and blue. The assignment of these two colors into the go and no-go condition was counterbalanced across participants. If the frame had the go color, participants had to press the space bar once as quickly as possible. If the frame had the no-go color, participants were told to not press any key. Instead, they should wait till the food image disappeared by itself. The cues thus indicated whether participants should make go or no-go responses.

Each time when participants pressed the space bar, the shopping cart switched from one lane to the other. When they did not respond, the cart remained on the same lane. We implemented this to independently manipulate the approach versus avoidance consequence as a result of a go or no-go response. The training thus consisted of four trial types, namely go-approach, go-avoidance, no-go-approach and no-go-avoidance (Figure 1). In the go-approach condition, the food always appeared on a different lane than the cart. Participants always had to respond, and as a result of their response, the cart switched to the same lane as the food, with the food eventually falling into the cart (i.e., the ‘approach’ consequence). In the go-avoidance condition, the food initially appeared on the same lane as the cart. After participants responded, the cart moved to the other lane, and the food eventually did not fall into the cart (i.e., the ‘avoidance’ consequence). In the no-go-approach (no-go-avoidance) condition, the food appeared on the same (different) lane than the cart. Participants did not respond, and the food images fell either into (no-go-approach) or outside (no-go-avoidance) the cart as a result. Participants were informed that the cart would move each time when they responded. However, no further explanation was given on what this meant in the instructions. Participants were further instructed that they could press the space bar at most once within one trial. If they were to press the space bar multiple times within one trial, only the first response was recorded, and the cart would also move only once. In both the go and no-go conditions, a food image disappeared when it nearly reached the bottom of a lane 2 seconds after image onset. The exposure time for each image was therefore matched on all trials. The inter-trial interval randomly varied between 1000 and 1500 milliseconds, in steps of 100 milliseconds.

Participants first started with a practice block. Four images were included in each of the four conditions, resulting in 16 images in total. Each image was randomly presented once. Food images used in the practice block were not used in the experimental blocks. Participants were asked to respond as accurately and quickly (in the case of go responses) as possible. No feedback was given on each trial. At the end of a practice block, the overall accuracy was computed. Participants were asked to practice again in case their accuracy was below 75%. They could practice at most 3 times (i.e., 3 practice blocks), after which the task proceeded to the experimental blocks even if the accuracy in the last practice block was still below 75%.

The four selected trained sets (i.e., except the untrained set) were used in the experimental blocks. Each food image was randomly presented once in each block, resulting in 32 trials per block. After every two blocks, the overall accuracy was computed and shown to participants. They could take a short break if necessary. The whole task consisted of 14 blocks, with 448 trials in total.

##### Rating After Training (∼3 Minutes).

After the training, participants received the rating task again. They were asked to report how attractive each presented food image appeared to them at that very moment. This time, only the 40 selected food images were used.

##### Memory Tasks (∼5 Minutes).

Participants then received two memory tasks. Note that they were not informed of the existence of the memory tasks before this part. The order of these two tasks were counterbalanced across participants. In the go/no-go memory task, they were asked to answer for each image, whether they had to press the space bar or not. The five options were: ‘sure did not press’, ‘maybe did not press’, ‘do not remember’, ‘maybe pressed’, ‘sure pressed’. In the approach/avoidance memory task, participants were asked whether each food eventually fell inside (i.e., approach) or outside (i.e., avoidance) the shopping cart. The five options were: ‘sure inside’, ‘maybe inside’, ‘do not remember’, ‘maybe outside’, ‘sure outside’. For the memory tasks, we included all 40 selected images, that is, including the 8 images from the untrained set. Since the untrained images were not used in the training, the correct answer for these images was ‘do not remember’. We included them for exploratory purposes to probe participants’ general tendency to select a certain option.

##### Rating After Memory (∼3 Minutes).

As an exploratory measure, participants received the same rating task again with the 40 selected food images after the memory tasks.

##### Questionnaires (∼5 Minutes).

Participant then filled out the 10-item restrained eating scale (Herman & Polivy, 1980). The total score varies between 0 and 35, with a larger score indicating a higher level of ‘restrained eating’. They then reported their height (in centimeters), weight (in kilos), how hungry they were (with a 9-point Likert scale, 1 = *Not at all*, 9 = *Very much*), and when they had their last meal (‘Less than 1 hour ago’, ‘1–3 hours ago’, ‘3–5 hours ago’, ‘More than 5 hours ago’, see Tzavella & Chambers, 2023). They were then debriefed, thanked, and received one course credit as compensation.

### Data Analysis

Data were analyzed using R (version 4.2.1; R Core Team, 2022), with the following R packages: afex (version 1.2.0; Singmann et al., 2022), bayesplot (version 1.10.0; Gabry & Mahr, 2022), bayestestR (version 0.13.0; Makowski et al., 2022), brms (version 2.18.0; Bürkner, 2022), cmdstanr (version 0.5.3; Gabry & Češnovar, 2022), ggpubr (version 0.6.0; Kassambara, 2023), kableExtra (version 1.3.4; Zhu, 2021), knitr (version 1.41; Xie, 2022), loo (version 2.5.1; Vehtari et al., 2022), MASS (version 7.3.58.1; Ripley, 2022), Rmisc (version 1.5.1; Hope, 2022), sjPlot (version 2.8.12; Lüdecke, 2022), tidybayes (version 3.0.2; Kay, 2022), and tidyverse (version 1.3.2; Wickham, 2022).

#### Data Exclusion.

Six participants restarted the experiment after having completed some trials, and finished the second attempt. Since the same food images were likely assigned into different training conditions in the two attempts, we decided to exclude the data from these participants. This exclusion criterion was *not* pre-registered. We further applied two pre-registered exclusion criteria. First, participants who had more than 10% of trials missing in either the pre-training rating task, the training task, or the rating task immediately following the training^2^ were excluded (1 participant). Since the experiment was administered online, data on some trials might not be registered due to technical issues (e.g., an unstable Internet connection). When missing trials occurred frequently, participants might have encountered technical issues during the experiment. We therefore adopted this criterion. Second, participants who had an accuracy 3 standard deviations below the sample mean *and* below 90% in any of the four conditions in the experimental blocks of the training were excluded (6 participants). The final sample consisted of 148 participants.

#### Pre-registered Data Analysis.

The main, pre-registered analyses focused on the changes in rating from before the training to immediately after training (i.e., the second rating task), in line with previous work (e.g., Chen et al., 2016). First, the mean ratings of the five selected sets before training were submitted to a repeated-measures ANOVA (one factor, five levels) to ensure that the average ratings were indeed matched prior to training. For each participant, the change in rating was then computed for each of the 40 selected images (i.e., Δrating = rating after training - rating before training). The mean Δrating was computed for the five selected sets for each participant. The mean Δratings from the four trained conditions (i.e., go-approach, go-avoidance, no-go-approach, and no-go-avoidance) were submitted to a 2 (response: go vs. no-go) by 2 (consequence: approach vs. avoidance) repeated-measures ANOVA.

We then used paired-samples *t* tests to examine simple effects within the trained conditions, and compared each trained condition against the untrained condition. To further examine the main effects of go and no-go responses, we combined the go-approach and the go-avoidance conditions into the go condition, and the no-go-approach and the no-go-avoidance conditions into the no-go condition. The go, no-go and untrained conditions were compared with each other with paired-samples *t* tests. We similarly examined the effects of approach and avoidance consequences, by combining the go-approach and no-go-approach conditions into the approach condition, and the go-avoidance and no-go-avoidance conditions into the avoidance condition. The approach, avoidance, and untrained conditions were compared with paired-samples *t* tests. The *p* values from the 14 paired-samples *t* tests were corrected for multiple comparisons using the Bonferroni method.

For all analyses, we used both the frequentist and Bayesian versions. For Bayesian repeated-measures ANOVA, the Bayes factor for each effect was computed across matched models. For Bayesian repeated-measures ANOVA and paired-samples *t* tests, the default prior settings in the R package *BayesFactor* (Morey & Rouder, 2022) and the software JASP (version 0.17.1; JASP Team, 2023) were used^3^. For Bayesian repeated-measures ANOVA, the priors were: r scale fixed effects = 0.5, r scale random effects = 1, and r scale covariates = 0.354. For Bayesian paired-samples *t* tests, the prior was a Cauchy distribution with a scale parameter of 0.707. For statistical inference, (corrected) *p* values were compared against the alpha level of 0.05. Bayes factors (*BF*_10_) quantified the likelihood of data under the alternative hypothesis against the null hypothesis. To interpret Bayes factors, we adopted verbal labels proposed in the literature (Wagenmakers et al., 2018). For effect sizes, we reported generalized eta-squared (*ges*) for ANOVA, and Cohen’s *d*_*z*_ and Hedges’ *g*_*av*_ for paired-samples *t* tests (Lakens, 2013).

### Results

#### Participant Characteristics.

After exclusion, 148 participants (124 females, 23 males, 1 non-binary; *M*_*age*_ = 18.82, *SD*_*age*_ = 1.84) remained in further analyses. One participant did not fill in the questionnaires at the end of the experiment. For the remaining 147 participants, the mean restraint eating score was 14.92 (*SD* = 5.75). Previous work has used a total score of 15 or higher on the restrained eating scale to indicate ‘restrained eating’ (e.g., Adams et al., 2019). Based on this criterion, 79 participants were classified as ‘restrained eaters’, and the remaining 68 as ‘non-restrained eaters’. Two participants did not report their height, weight, hunger, and the time of last meal. For the remaining 146 participants, the mean body mass index is 21.95 (*SD* = 3.47, *Min* = 16.54, *Max* = 44.98) and the mean hunger level was 4.71 (*SD* = 2.33). 21.23% of the participants had their last meal less than 1 hour ago, 44.52% 1–3 hours ago, 23.29% 3–5 hours ago, and the remaining 10.96% more than 5 hours ago.

#### Performance in the Training (Not Pre-registered).

**Figure 2.**
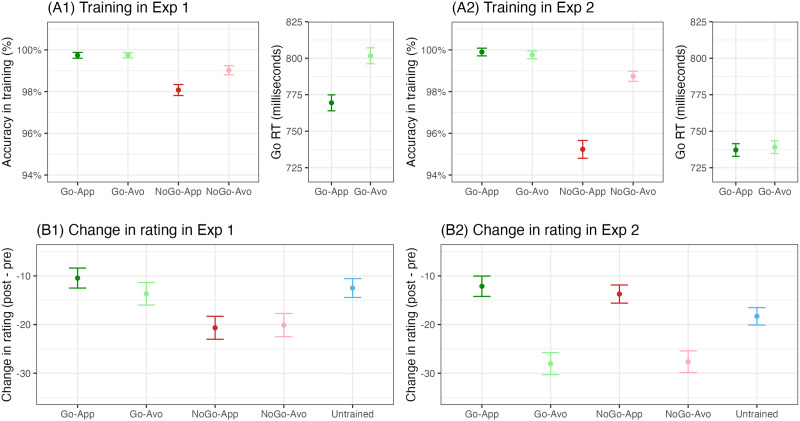
Performance in the training in Experiment 1 (A1) and 2 (A2), and change in rating in Experiment 1 (B1) and 2 (B2). The error bars stand for 95% within-subject confidence intervals. App = Approach, Avo = Avoidance.

Participants could practice up to 3 times before they proceeded to the experimental blocks in the training. Only one participant did not meet the 75% accuracy criterion after 3 practice blocks, but their accuracy in the experimental blocks was high, so we also retained their data. Participants overall performed accurately in the experimental blocks: go-approach *M* = 99.74%, *SD* = 0.83%, go-avoidance *M* = 99.74%, *SD* = 0.66%, no-go-approach *M* = 98.08%, *SD* = 1.92%, and no-go-avoidance *M* = 99.02%, *SD* = 1.31% (Figure 2-A1). A 2 (response: go vs. no-go) by 2 (consequence: approach vs. avoidance) repeated-measures ANOVA on accuracy showed that the main effect of go versus no-go response (*F*(1, 147) = 158.34, *p* < .001, *ges* = 0.180, *BF*_10_ = 2.12 × 10^20^), the main effect of approach versus avoidance consequence (*F*(1, 147) = 23.22, *p* < .001, *ges* = 0.034, *BF*_10_ = 1000.4), and the interaction effect (*F*(1, 147) = 21.74, *p* < .001, *ges* = 0.034, *BF*_10_ = 6.45 × 10^4^) were all statistically reliable. Pairwise comparisons (Table 1) showed that participants were more accurate on go trials than on no-go trials when the consequence was matched (see Go-App vs. NoGo-App and Go-Avo vs. NoGo-Avo in Table 1). Furthermore, while the approach versus avoidance consequence did not modulate go accuracy, participants were more accurate on no-go-avoidance trials than on no-go-approach trials. Comparison of the mean go response times (RTs) on correct trials showed that participants were faster on go-approach trials (*M* = 769.5, *SD* = 122.4) than on go-avoidance trials (*M* = 801.7, *SD* = 143.1). Overall, the approach consequence facilitated go responses (as reflected in go RTs) while the avoidance consequence facilitated no-go responses (as reflected in the accuracy).

**Table 1.** Pairwise comparisons on the performance in the training in Experiments 1 and 2.

Comparison	*diff*	*lowerCI*	*upperCI*	*df*	*t*	*p*	*BF*_10_	*d*_*z*_	*g*_*av*_
*Accuracies (%) in the training in Experiment 1*									
Go-App vs. Go-Avo	<0.001	−0.129	0.129	147	<0.01	1.000	9.15 × 10^−2^	<0.001	<0.001
NoGo-App vs. NoGo-Avo	−0.947	−1.321	−0.574	147	−5.01	<.001	8.16 × 10^3^	0.412	0.586
Go-App vs. NoGo-App	1.665	1.355	1.976	147	10.60	<.001	6.57 × 10^16^	0.871	1.210
Go-Avo vs. NoGo-Avo	0.718	0.485	0.951	147	6.09	<.001	1.05 × 10^6^	0.500	0.728

*Go response times (in milliseconds) in the training in Experiment 1*									
Go-App vs. Go-Avo	−32.20	−39.92	−24.49	147	−8.25	<.001	8.13 × 10^10^	0.678	0.242

*Accuracies (%) in the training in Experiment 2*									
Go-App vs. Go-Avo	0.136	0.043	0.228	157	2.89	.017	4.88	0.230	0.250
NoGo-App vs. NoGo-Avo	−3.504	−4.020	−2.988	157	−13.41	<.001	4.39 × 10^24^	1.067	1.439
Go-App vs. NoGo-App	4.668	4.161	5.176	157	18.16	<.001	1.19 × 10^37^	1.445	2.562
Go-Avo vs. NoGo-Avo	1.029	0.759	1.298	157	7.54	<.001	2.15 × 10^9^	0.600	0.890

*Go response times (in milliseconds) in the training in Experiment 2*									
Go-App vs. Go-Avo	−1.93	−8.10	4.23	157	−0.62	.537	0.107	0.049	0.025

*Note*. For the analyses on accuracies in Experiments 1 and 2, p values were corrected for multiple comparisons using the Bonferroni method separately. App = Approach, Avo = Avoidance.

#### Change in Rating Immediately After Training (Pre-registered).

The average ratings for the five selected sets of images were closely matched before training (Go-App: *M* = 57.37, *SD* = 18.18; Go-Avo: *M* = 57.58, *SD* = 17.99; NoGo-App: *M* = 57.37, *SD* = 18.05; NoGo-Avo: *M* = 57.54, *SD* = 18.27; Untrained: *M* = 57.58, *SD* = 18.12). Repeated-measures ANOVA showed that the effect of food condition was not statistically significant, *F*(3.79, 557.14) = 1.39, *p* = .239, *ges* < 0.001.

We then computed changes in rating from before the training to immediately after training (i.e., Δrating) for the selected images (see panel B1 in Figure 2). Note that on average the ratings all decreased, which was likely due to the phenomenon of regression to the mean. That is, since we selected items that received the highest ratings before the training, the ratings for these items tended to ‘regress’ to the average when measured the second time (e.g., participants might tend to use the full range of the scale). Such general decreases in ratings have been observed repeatedly in previous work when highly appetitive items were rated twice (Chen et al., 2016, 2018). The inclusion of untrained items as a baseline allowed us to isolate changes in rating that were specifically due to the training.

Repeated-measures ANOVA on changes in rating with response (go vs. no-go) and consequence (approach vs. avoidance) as factors showed a main effect of response (*F*(1, 147) = 46.96, *p* < .001, *ges* = 0.054, *BF*_10_ = 4.695 × 10^7^) and no main effect of consequence (*F*(1, 147) = 0.65, *p* = .420, *ges* < 0.001, *BF*_10_ = 0.149). The interaction effect was statistically significant (*F*(1, 147) = 4.97, *p* = .027, *ges* = 0.003), but the Bayes factor was inconclusive (*BF*_10_ = 1.488).

To further examine how the different training conditions influenced stimulus evaluation, we conducted a series of planned comparisons (Table 2). First, we compared the go-approach condition with the no-go-approach condition, and the go-avoidance condition with the no-go-avoidance condition. In both cases, the rating decreased more from before to after the training for no-go items compared to go items, when the consequences were matched. The difference between go-approach and go-avoidance was inconclusive, while the Bayes factor supported the null hypothesis for the comparison between no-go-approach and no-go-avoidance. Note that descriptively the effects of consequence were in opposite directions for the go and no-go conditions, which explained the interaction effect observed above.

**Table 2.** Pairwise comparisons on changes in ratings (immediately after training) in Experiment 1.

Comparison	*diff*	*lowerCI*	*upperCI*	*df*	*t*	*p*	*BF*_10_	*d*_*z*_	*g*_*av*_
*Comparisons among the trained conditions*									
Go-App vs. NoGo-App	10.44	7.51	13.37	147	7.04	<.001	1.21 × 10^8^	0.579	0.582
Go-Avo vs. NoGo-Avo	6.73	3.72	9.74	147	4.42	<.001	739.3	0.363	0.375
Go-App vs. Go-Avo	2.67	0.07	5.26	147	2.03	0.617	0.673	0.167	0.159
NoGo-App vs. NoGo-Avo	−1.04	−3.60	1.51	147	−0.81	1.000	0.126	0.066	0.055

*Comparisons against the untrained condition*									
Go-App vs. Untrained	2.45	−0.05	4.96	147	1.94	0.768	0.562	0.159	0.175
Go-Avo vs. Untrained	−0.21	−2.86	2.43	147	−0.16	1.000	0.093	0.013	0.015
NoGo-App vs. Untrained	−7.98	−10.74	−5.23	147	−5.72	<.001	1.89 × 10^5^	0.471	0.507
NoGo-Avo vs. Untrained	−6.94	−9.71	−4.18	147	−4.96	<.001	6.61 × 10^3^	0.408	0.458

*Comparisons for the main effects of Go/NoGo*									
Go vs. NoGo	8.58	6.11	11.06	147	6.85	<.001	4.62 × 10^7^	0.563	0.534
Go vs. Untrained	1.12	−1.11	3.35	147	0.99	1.000	0.148	0.082	0.084
Untrained vs. NoGo	7.46	5.02	9.91	147	6.03	<.001	7.79 × 10^5^	0.495	0.511

*Comparisons for the main effects of Approach/Avoidance*									
App vs. Avo	0.81	−1.17	2.79	147	0.81	1.000	0.126	0.067	0.052
App vs. Untrained	−2.77	−4.95	−0.58	147	−2.50	0.190	1.84	0.205	0.202
Untrained vs. Avo	3.58	1.33	5.83	147	3.14	0.028	10.04	0.258	0.264

*Note*. P values were corrected for multiple comparisons using the Bonferroni method. App = Approach, Avo = Avoidance.

Next, we compared the four trained conditions against the untrained condition. The ratings decreased more for both no-go-approach and no-go-avoidance items compared to untrained items. The difference between go-approach and untrained items was inconclusive, while the difference between go-avoidance and untrained items received support for the null hypothesis. To further examine the main effects of go/no-go responses, we combined the go-approach and go-avoidance conditions into the go condition, and the no-go-approach and no-go-avoidance conditions into the no-go condition. Overall, the ratings for no-go items decreased more than both go and untrained items, while no difference was observed between the latter two conditions. Similar examination of the main effects of approach/avoidance consequences showed no difference between the approach and avoidance conditions. The difference between the approach and the untrained condition was inconclusive, while the ratings for the avoidance condition decreased more than the untrained condition. However, as the comparisons above have shown, this difference was mainly driven by the no-go-avoidance condition, but not the go-avoidance condition.

### Discussion

Using a novel training that combined go/no-go and approach/avoidance actions orthogonally, we were able to replicate the effect of GNG on stimulus evaluation (Chen et al., 2016) when the cues indicated go and no-go actions. Immediately after the training, no-go items were evaluated less positively than go and untrained items, while no difference was observed between the latter two conditions. Approach/avoidance consequences overall did not influence stimulus evaluation.

One explanation for the absence of an approach/avoidance effect might be that participants simply ignored the consequences of their responses in the training. After all, the cues directly indicated to them whether they should respond or not. The consequence of one’s response was therefore irrelevant for performing the task. However, this did not seem to be the case. Participants made go responses more quickly if the go responses led them to approach a food than if the go responses led them to avoid a food. Related, they were more accurate in not responding if not responding led them to avoid a food than if not responding led them to approach a food. Approach consequences thus facilitated go responses, while avoidance consequences facilitated no-go responses. Previous work has shown that go responses are associated with positive valence (reward), whereas no-go responses are associated with negative valence (punishment; Guitart-Masip et al., 2014). Since approaching appetitive foods may be positive, while avoiding appetitive foods may be negative, the shared valence between go responses and approach, and between no-go responses and avoidance, may explain why approach/avoidance consequences influenced go/no-go responses here. The exact mechanism for this observation notwithstanding, this finding indicates that participants incorporated the consequence information while executing go and no-go responses in the training. Immediately after the training, participants received two memory tasks to probe their memory of whether each item was associated with go and no-go response, and with approach and avoidance consequence, respectively. We report the results on memory performance in the Supplemental Materials. To preview some of the results, participants in Experiment 1 could to some extent discriminate between approach and avoidance items when asked to report whether each item was associated with approach or avoidance consequence. This finding reinforces the notion that the absence of an approach/avoidance effect on stimulus evaluation could not be explained by participants simply ignoring the consequences of their responses.

An alternative explanation for the absence of an approach/avoidance effect may be that seeing a food item falling inside or outside the shopping cart (i.e., how we defined approach and avoidance consequence here) might simply not be enough to change stimulus evaluation, or that it might not be enough when the interpretation of these actions as approach/avoidance is hindered because the meaning of these consequences is not specified in the instructions. We carried out Experiment 2 to test these possibilities.

## EXPERIMENT 2

We used the same experimental procedure in Experiment 2. The only difference from Experiment 1 was that in the training, we now changed the cues to indicate to participants whether they should approach or avoid certain food items, rather than whether they should respond or not. We expected an effect of approach versus avoidance on stimulus evaluation, thus ruling out the alternative explanation discussed above (i.e., that our implementation of approach/avoidance actions could not change stimulus evaluation). More importantly, the main question of interest was whether we would still observe a no-go devaluation effect in Experiment 2, when the same responses were now *not* framed as go and no-go actions. We did not have a directional hypothesis for the GNG effect.

### Methods

#### Sample Size.

We similarly planned to recruit 160 participants. Sensitivity analysis in G*Power (version 3.1.9.6; Faul et al., 2007) shows that with 160 participants, we have 80% power to detect effect sizes as small as Cohen’s *d*_*z*_ of 0.223 (with a two-sided paired-samples *t* test and an alpha level of .05). For AAT effects on explicit stimulus evaluation, there was no published meta-analysis at the time of conducting Experiment 2. We therefore conducted a meta-analysis of 3 well-powered online studies examining AAT effects with the manikin procedure in the context of food stimuli (Smith et al., 2020). This meta-analysis found that the overall AAT effect on explicit stimulus evaluation was Hedges’ *g* = 0.401, which was larger than the effect size from the sensitivity analysis. Our sample size thus provides sufficient statistical power to detect the AAT effect on explicit stimulus evaluation.

#### Participants.

Participants who had participated in Experiment 1 could not sign up for Experiment 2. While testing, we noticed that some participants were younger than 18 years old, which was the minimum age to participate as specified in the informed consent. Note that none of the participants in Experiment 1 was younger than 18 years old, which was the reason why we did not explicitly mention this as an exclusion criterion in the pre-registrations. However, since these participants needed course credits for their study, we allowed them to participate, but immediately deleted their data after they finished (8 participants). In addition to these 8 participants, 163 participants (143 females, 19 males, 1 did not report their gender; *M*_*age*_ = 18.90, *SD*_*age*_ = 2.46) took part in the experiment for course credit in November, 2023. Note that we exceeded the planned sample size, because we requested extra credits to recruit more participants, to leave some room for excluding participants who were younger than 18 years old.

#### Apparatus, Materials and Procedure.

The same apparatus and materials as in Experiment 1 were used in Experiment 2. The only difference was that the cues in the training now indicated whether participants should approach or avoid a certain food item (Figure 1). More concretely, participants were told that when a food image was surrounded by a certain color (i.e., the approach cue), they should bring the cart on the same lane as the food, so that the food would eventually fall into the cart. In contrast, when a food image was surrounded by a different color (i.e., the avoidance cue), they should bring the cart on a different lane than the food, so that the food would not fall into the cart. The assignment of the two colors into the approach and avoidance cues was counterbalanced across participants. As in Experiment 1, we used an orthogonal manipulation of go vs. no-go responses and approach vs. avoidance consequences. Half of the time, the cart was already on the correct lane so participants did not need to respond, whereas the other half of the time the cart was on the incorrect lane so they needed to press the space bar. The rest of the procedure was the same as in Experiment 1.

### Data Analysis

The same data analysis approach as in Experiment 1 was used.

### Results

#### Participant Characteristics.

In total, 5 participants were excluded based on the three pre-registered exclusion criteria: (1) restarting the experiment after having completed some trials (5 participants), (2) having more than 10% of the trials missing in either the first rating task, the training, or the rating task immediately after training (0 participant), and (3) having an accuracy 3 standard deviations below the sample mean and below 90% in any of the four conditions in the training (0 participant). 158 participants remained in further analyses (138 females, 19 males, 1 did not report their gender; *M*_*age*_ = 18.85, *SD*_*age*_ = 2.36; *M*_*restraint*_ = 14.39, *SD*_*restraint*_ = 6.43, 70 classified as ‘restrained eaters’, and 88 as ‘non-restrained eaters’). Three participants did not report their height and/or weight. For the remaining 155 participants, the mean body mass index was 21.77 (*SD* = 3.47, *Min* = 15.7, *Max* = 34.72). Two participants did not report their hunger level or the time of last meal. For the remaining 156 participants, the mean hunger level was 4.74 (*SD* = 2.55). Approximately 15.38% of the participants had their last meal less than 1 hour ago, 53.21% 1–3 hours ago, 16.03% 3–5 hours ago, and 15.38% more than 5 hours ago. Direct comparisons between Experiments 1 and 2 revealed no statistically significant differences in these sample characteristics (see the Supplemental Materials).

#### Performance in the Training (Not Pre-registered).

One participant did not meet the 75% accuracy criterion after 3 practice blocks (but had good accuracy in the experimental blocks). The overall accuracy in the experimental blocks was again high: go-approach *M* = 99.90%, *SD* = 0.40%, go-avoidance *M* = 99.76%, *SD* = 0.68%, no-go-approach *M* = 95.23%, *SD* = 3.23%, and no-go-avoidance *M* = 98.73%, *SD* = 1.63%. In line with Experiment 1, repeated-measures ANOVA showed that the main effect of go versus no-go response (*F*(1, 157) = 334.98, *p* < .001, *ges* = 0.373, *BF*_10_ = 9.17 × 10^35^), the main effect of approach versus avoidance consequence (*F*(1, 157) = 165.76, *p* < .001, *ges* = 0.172, *BF*_10_ = 1.28 × 10^18^), and the interaction effect (*F*(1, 157) = 182.95, *p* < .001, *ges* = 0.196, *BF*_10_ = 1.73 × 10^36^) were all statistically reliable. Pairwise comparisons (Table 1) showed that participants were more accurate on go trials than on no-go trials when the consequence was matched (see Go-App vs. NoGo-App and Go-Avo vs. NoGo-Avo in Table 1). Different from Experiment 1 though, the approach versus avoidance consequence modulated the accuracy on both go and no-go trials. Participants were more accurate on go-approach than go-avoidance trials, and more accurate on no-go-avoidance than no-go-approach trials. Another difference from Experiment 1 was that there was no statistically reliable difference in go RTs between go-approach (*M* = 737.1, *SD* = 79.9) and go-avoidance trials (*M* = 739.1, *SD* = 76.9). Participants in Experiment 2 overall responded more quickly than those in Experiment 1 (compare panel A1 and A2 in Figure 2). This speed-accuracy trade-off may explain why we observed an effect of consequence only on go RTs in Experiment 1 and only on go accuracy in Experiment 2. Overall, we thus again observed that approach consequence facilitated go responses and avoidance consequence facilitated no-go responses, although both were now reflected in the accuracy.

#### Change in Rating Immediately After Training (Pre-registered).

The average ratings for the five selected sets of images were again closely matched before the training (Go-App: *M* = 57.76, *SD* = 19.09; Go-Avo: *M* = 57.77, *SD* = 19.19; NoGo-App: *M* = 57.82, *SD* = 19.12; NoGo-Avo: *M* = 57.97, *SD* = 19.08; Untrained: *M* = 57.86, *SD* = 19.09). Note that these average ratings were also very close to those observed in Experiment 1. Repeated-measures ANOVA showed that the effect of food condition was not statistically significant, *F*(3.88, 609.56) = 0.81, *p* = .517, *ges* < 0.001.

We similarly computed the changes in rating from before to after the training, and submitted them to a 2 (response: go vs. no-go) by 2 (consequence: approach vs. avoidance) repeated-measures ANOVA. We observed a significant main effect of approach versus avoidance consequence (*F*(1, 157) = 109.58, *p* < .001, *ges* = 0.125, *BF*_10_ = 5.905 × 10^16^), no main effect of go versus no-go response (*F*(1, 157) = 0.56, *p* = .456, *ges* < 0.001, *BF*_10_ = 0.123), and also no interaction effect (*F*(1, 157) = 1.19, *p* = .278, *ges* < 0.001, *BF*_10_ = 0.238). Planned comparisons showed that when the consequence was matched (Go-App vs. NoGo-App and Go-Avo vs. NoGo-Avo in Table 3), there was no effect of go and no-go response on stimulus evaluation. Instead, in line with our prediction, when go and no-go response was matched (Go-App vs. Go-Avo and NoGo-App vs. NoGo-Avo in Table 3), the ratings for avoided items decreased more than approached items. Compared with the untrained baseline, the ratings for go-approach and no-go-approach items decreased less, while the ratings for go-avoidance and no-go-avoidance decreased more after the training. Lastly, we examined the main effects of go/no-go responses and approach/avoidance consequences. We found no overall difference between go and no-go items, and the comparisons against the untrained condition revealed inconclusive evidence (although both were in the direction of supporting the null hypothesis). For approach/avoidance effects, we found that the evaluation for avoided items decreased more than untrained items, which in turned decreased more than approached items (see panel B2 in Figure 2).

**Table 3.** Pairwise comparisons on changes in ratings (immediately after training) in Experiment 2.

Comparison	*diff*	*lowerCI*	*upperCI*	*df*	*t*	*p*	*BF*_10_	*d*_*z*_	*g*_*av*_
*Comparisons among the trained conditions*									
Go-App vs. NoGo-App	1.60	−0.67	3.87	157	1.40	1.000	0.229	0.111	0.099
Go-Avo vs. NoGo-Avo	−0.39	−2.95	2.16	157	−0.30	1.000	0.093	0.024	0.017
Go-App vs. Go-Avo	15.90	12.39	19.41	157	8.94	<.001	5.87 × 10^12^	0.711	0.818
NoGo-App vs. NoGo-Avo	13.90	10.74	17.07	157	8.67	<.001	1.25 × 10^12^	0.690	0.707

*Comparisons against the untrained condition*									
Go-App vs. Untrained	6.17	3.79	8.55	157	5.12	<.001	1.36 × 10^4^	0.408	0.372
Go-Avo vs. Untrained	−9.73	−12.73	−6.72	157	−6.39	<.001	5.12 × 10^6^	0.509	0.485
Go-Avo vs. Untrained	−9.73	−12.73	−6.72	157	−6.39	<.001	5.12 × 10^6^	0.509	0.485
NoGo-App vs. Untrained	4.57	2.13	7.01	157	3.69	0.004	55.12	0.294	0.272
NoGo-Avo vs. Untrained	−9.33	−12.33	−6.33	157	−6.15	<.001	1.53 × 10^6^	0.489	0.466

*Comparisons for the main effects of Go/NoGo*									
Go vs. NoGo	0.60	−0.99	2.20	157	0.75	1.000	0.117	0.059	0.036
Go vs. Untrained	−1.78	−3.84	0.29	157	−1.70	1.000	0.362	0.135	0.106
Untrained vs. NoGo	2.38	0.15	4.61	157	2.11	0.511	0.766	0.168	0.139

*Comparisons for the main effects of Approach/Avoidance*									
App vs. Avo	14.90	12.09	17.71	157	10.47	<.001	5.53 × 10^16^	0.833	0.830
App vs. Untrained	5.37	3.24	7.50	157	4.98	<.001	7.50 × 10^3^	0.397	0.339
Untrained vs. Avo	9.53	6.81	12.25	157	6.93	<.001	7.89 × 10^7^	0.551	0.494

*Note*. P values were corrected for multiple comparisons using the Bonferroni method. App = Approach, Avo = Avoidance.

### Discussion

By changing the cues in the training to indicate whether participants should approach or avoid certain food items, we observed an approach/avoidance effect on stimulus evaluation in Experiment 2. The absence of such an effect in Experiment 1 therefore could not be explained by our operationalization of approach versus avoidance (i.e., foods falling inside or outside a shopping cart) being ineffective. More importantly, go and no-go responses did not influence stimulus evaluation in Experiment 2, even though the motor responses made in the training were the same in both experiments. We again observed that approach facilitated go responses and avoidance facilitated no-go responses in the training, although now both effects were observed in the accuracy but not in the go RT.

## EXPLORATORY COMPARISONS BETWEEN EXPERIMENTS 1 AND 2

In an exploratory analysis, we directly compared the two experiments. For each participant, we computed an overall GNG effect, as the difference in changes in ratings between the go conditions (i.e., go-approach and go-avoidance) and the no-go conditions (i.e., no-go-approach and no-go-avoidance). An AAT effect was similarly computed as the difference in changes in ratings between the approach conditions (i.e., go-approach and no-go-approach) and the avoidance conditions (i.e., go-avoidance and no-go-avoidance).

The GNG effect was larger in Experiment 1 than in Experiment 2, *diff* = 7.98, 95% *CI* = [5.04, 10.92], *t*(253.8) = 5.35, *p* < .001, *BF*_10_ = 9.44 × 10^4^, Hedge’s *g*_*av*_ = 0.611. The AAT effect was larger in Experiment 2 than in Experiment 1, *diff* = 14.09, 95% *CI* = [10.66, 17.52], *t*(278.3) = 8.09, *p* < .001, *BF*_10_ = 2.14 × 10^11^, Hedge’s *g*_*av*_ = 0.923. For exploratory reasons, we also compared the AAT effect in Experiment 2 with the GNG effect in Experiment 1, and found the former to be larger than the latter, *diff* = 6.32, 95% *CI* = [2.59, 10.05], *t*(301.3) = 3.33, *p* = .001, *BF*_10_ = 22.45, Hedge’s *g*_*av*_ = 0.380. Lastly, we compared the AAT effect in Experiment 1 with the GNG effect in Experiment 2, and found no significant difference, *diff* = 0.21, 95% *CI* = [−2.33, 2.74], *t*(286.8) = 0.16, *p* = .872, *BF*_10_ = 0.128, Hedge’s *g*_*av*_ = 0.018.

In addition to the exploratory analyses reported here, we conducted further exploratory analyses to examine (1) performance in the training task over time, (2) the potential influences of cue colors (i.e., blue versus green) on both training performance and training-induced changes in stimulus evaluation, (3) performance in the memory tasks, (4) relations between memory and training effects on evaluation, and (5) ratings after the memory tasks. Interested readers can find the results of these additional exploratory analyses in the Supplemental Materials.

## GENERAL DISCUSSION

In the current research, we examined the role of action execution and action interpretation in the evaluative influences of go/no-go and approach/avoidance actions. In a novel training that combined both go/no-go and approach/avoidance actions orthogonally, participants either responded or did not respond, to either approach or avoid certain food items. Despite making the same motor responses, when the cues indicated whether participants should execute go or no-go actions (Experiment 1), we observed a GNG effect, but no AAT effect; instead, when the cues indicated whether participants should execute approach or avoidance actions (Experiment 2), we observed an AAT effect, but no GNG effect.

### Action Execution and Action Interpretation in the GNG and AAT Effects

We found that the same motor responses could be interpreted as either go/no-go or approach/avoidance actions, which in turn determined the effects of the training on stimulus evaluation. The recent work by Houben (2023) showed that the same go/no-go responses could be interpreted as actions with different evaluative meanings (‘take’ and ‘do not take’ versus ‘throw away’ and ‘keep’), which also influenced the effect of GNG on stimulus evaluation. Both sets of findings highlight the role of action interpretation in evaluative changes induced by simple motor responses. As such, our results can be seen as a conceptual replication of Houben (2023). The current findings are thus in line with theoretical accounts that emphasize the role of action interpretation in determining the evaluative influences of motor responses (i.e., the common coding account and the inferential account), and argue against accounts that view action execution per se as the origin of the evaluative influences of motor responses (i.e., the BSI theory, the devaluation-by-no-go account, and the motivational systems account). Some recent theorizing on the GNG appears to have incorporated a role of action interpretation (although this was not explicitly discussed), for instance by proposing that the GNG influences stimulus evaluation via a value-updating process instigated by the *decisions* to either act or not act on a certain (food) stimulus (Veling et al., 2022). Our results add to this idea, by showing that interpreting the trained responses as go and no-go actions (or in the terminology used by the authors of this account, action and inaction decisions; Veling et al., 2022) is likely a prerequisite for the assumed value-updating process. More broadly, by revealing the similarity between the GNG and AAT effects in a combined training task, the current results suggest that some common cognitive processes may underlie both effects, in which action interpretation plays a crucial role.

One question raised by the current findings is why no-go and avoidance actions are sometimes interpreted as negative actions, and approach (and sometimes go) actions are sometimes interpreted as positive actions. In line with Houben (2023), we speculate that one possibility may be that the former actions may be interpreted as ‘not taking’ or ‘rejecting’ something, whereas the latter actions may be interpreted as ‘taking’ or ‘keeping’ something, which then imported evaluative meanings to the actions. This may explain why the AAT effect in Experiment 2 was larger than the GNG effect in Experiment 1. That is, in the ‘food shopping’ game, we operationalized approach as food items falling into the shopping cart, and avoidance as food items falling outside the shopping cart. Food items falling inside or outside one’s shopping cart may lead participants to easily interpret these actions as ‘taking’ or ‘rejecting’ something, which has clear evaluative meanings in the current context. In contrast, while participants might also interpret go actions as ‘taking’ something and no-go actions as ‘not taking’ something (Houben, 2023), such interpretations may be more ambiguous than the approach/avoidance actions in the current shopping cart situation. As such, the approach/avoidance actions in Experiment 2 led to larger changes in stimulus evaluation than the go/no-go actions in Experiment 1.

Action execution in itself does not seem sufficient to induce evaluative changes. Instead, motor responses need to be interpreted as actions with evaluative connotations. This raised the question of whether action execution is even necessary for changing stimulus evaluation. This question is theoretically relevant, because the common coding account and the inferential account provide different answers to this question. As mentioned above, both accounts are in line with the current findings. Our current results thus do not allow us to distinguish between these two accounts. However, the inferential account makes the unique prediction that evaluative changes induced by motor responses are driven by the cognitive inferences that people make, and such cognitive inferences may be made even when people do not actually execute the responses (Van Dessel et al., 2019). Supporting the inferential account, previous work has shown that telling participants that they were going to approach certain stimuli and avoid certain stimuli in task instructions was sufficient to change their evaluation of these stimuli, without them actually executing approach and avoidance actions (Smith et al., 2020; Van Dessel et al., 2015; Van Dessel, Eder, & Hughes, 2018). This instruction-based AAT effect was mainly observed for initially more neutral, unfamiliar stimuli, but not highly familiar stimuli for which people already had strong likes and dislikes prior to training (Van Dessel et al., 2015, 2020). This finding has been related to the diagnosticity of approach/avoidance actions compared to other information for making evaluative inferences and giving evaluative responses (Van Dessel, De Houwer, Gast, et al., 2016). For the GNG, in Experiment 5 by Chen et al. (2016), participants observed a GNG with appetitive food stimuli without actually making any responses. No effect on stimulus evaluation was found after merely observing the GNG. Together, these findings suggest that action execution may not be needed to change the evaluation of neutral stimuli, but may still be necessary for highly valenced stimuli (such as appetitive food items used here). Repeatedly executing responses may provide more opportunities to interpret these responses as valenced actions. Furthermore, engaging in a training task may increase the relevance of the trained actions compared to merely observing a training, making them more diagnostic for evaluation. Both factors may contribute to the effectiveness of a training, making action execution still an important component for changing the evaluation of valenced stimuli. Lastly, different learning processes as proposed by the common coding account and the inferential account may be engaged (or be engaged to different extent) depending on the initial valence of a stimulus and whether participants execute motor responses or not. Here we used only highly appetitive items, to be in line with previous work on the GNG (Chen et al., 2016), and given the interest in using the GNG and AAT to reduce the evaluation of appetitive items in applied settings. Future work can use a broader range of stimuli (e.g., highly appetitive and less appetitive items), to further test the roles of action execution and action interpretation in response-induced evaluative changes for these stimuli.

### Practical Implications, Limitations, and Future Directions

Both the GNG and AAT are increasingly being used as cost-effective behavior change tools in various health domains. In line with Houben (2023), our results suggest that to maximize the effectiveness of such trainings, it is important to disambiguate how people frame and interpret their motor responses. Action framing should preferably carry clear and strong evaluative meanings, which may be achieved in many ways. For instance, motor responses may be labeled as certain valenced actions via task instructions (Houben, 2023), by using visual feedback that helps disambiguate action interpretation (e.g., such as food items falling into or outside a shopping cart as used here), and by providing further consequences of actions in the training (e.g., an avatar becoming sick after approaching unhealthy food items, see Van Dessel, Hughes, & De Houwer, 2018) etc. These elements may be further combined to make such trainings potentially more effective.

Before these suggestions are implemented in applied settings, several limitations and open questions from the current research need to be further examined. First, although previous research has shown that go/approach are generally perceived to be positive, and no-go/avoidance to be negative actions (Eder & Rothermund, 2008; McCulloch et al., 2012), we did not measure participants’ evaluation of these action labels here. Individuals may differ in how positive/negative they perceive these action labels to be, and it would be useful to know whether one’s evaluation of these action labels is related to the magnitude of the training effect (e.g., Van Dessel, Hughes, & De Houwer, 2018). It would also be useful to know whether the evaluation of these action labels is related to how people interpret these actions (e.g., as ‘taking’ and ‘not taking’ something), as we speculated above.

Second, we only examined the effects of GNG and AAT on explicit stimulus evaluation, which might raise the concern that our results were driven by experimenter demand. Demand-related processes have been proposed as one explanation of the GNG and AAT effects. According to the inferential account, demand-related inferences may be one of the cognitive inferences that people make during training (Van Dessel et al., 2019). For instance, it is possible that participants may infer that food items falling inside or outside the shopping cart implies that the researchers want them to change their ratings in accordance with this effect, and they change their ratings accordingly. Such demand-related inferences are a valid explanation of GNG and AAT effects, and might even be of relevance to achieve success with these procedures. Importantly, however, since the instructions did not specify the valence of any responses, such demand-related effects would still depend on action interpretation (i.e., participants need to first interpret their actions as valenced along a dimension indicated by action framing, before they can make demand-related inferences and provide compatible responses), and thus still support the main conclusion of the current research. Although demand may play a role, it is unlikely that the current results are completely driven by demand, for two main reasons. First, we used the same experimental procedure from previous work on GNG training effects (Chen et al., 2016). Both the overall pattern and magnitude of the current results closely match those from previous work (Chen et al., 2016), suggesting that the same cognitive processes are likely involved. Importantly, previous work has gone beyond stimulus evaluation, and used measures that are less susceptible to demand. For instance, both the GNG and AAT have been shown to influence food choices for real consumption (e.g., Chen et al., 2019, 2021; Porter et al., 2018; Veling et al., 2021; Wu et al., 2023), which cannot be explained by demand compliance alone. Furthermore, changes in preference were correlated with changes in ratings (Johannes et al., 2021; Veling et al., 2021), suggesting that common cognitive processes (e.g., changes in stimulus value) may underlie both behavioral effects. Second, purely complying with demand to adjust one’s ratings would be cognitively very demanding in the current experiments. We used a large number of images in the rating tasks (90 before the training, and 40 after the training), and the two rating tasks were 30 minutes apart from each other. If participants were purely adjusting their ratings to comply with demand, they would have to (1) roughly remember their rating for each image from about 30 minutes ago, (2) remember the condition of an image in the training, and (3) have precise knowledge of what we as researchers expected (e.g., for Experiment 1, we expected decreased evaluations for no-go items only, but no changes in evaluations for go items compared to untrained items), and (4) adjust their ratings accordingly to yield effect sizes in line with previous work. It seems unlikely that participants would be willing or able to do this. We observed that on average participants took less than 3 seconds to rate an image (this included the time of clicking on the ‘Continue’ button beneath the slider, so the actual time of rating an image was even shorter). This makes it unlikely that they were engaging in such complex demand-related processes to give their ratings. Instead, it is more likely that they were rating the images based on how attractive each image appeared to them at that very moment, which is cognitively much less demanding and in line with the instructions (a more direct cue of experimenter demand). Together, these observations suggest that experimenter demand is unlikely to completely explain the current results. However, for follow-up research, it can be important to go beyond stimulus evaluation, and examine whether action interpretation would similarly influence the effects of GNG and AAT on other behavioral outcomes (such as consequential food choices).

We used cues to make people interpret the same motor responses as either go/no-go or approach/avoidance actions, but not both. One intriguing question is whether people could simultaneously interpret their responses as go/no-go *and* approach/avoidance actions, and whether this ‘double’ action interpretation would have additive effects on stimulus evaluation compared to action interpretation on one dimension alone. For instance, participants may be instructed to respond to approach a stimulus, and to not respond to avoid a stimulus, and be made aware of both features of their actions. If this training yields stronger effects than when the cues emphasize only one dimension, such a training would be a good tool to use for practical purposes, to induce stronger and potentially more long-lasting behavior change.

In the current investigation, we focused on the GNG and AAT, as two popular tasks that use simple motor responses to induce behavior change. However, as alluded to earlier, other tasks also exist. For instance, in the cue-approach training (CAT), participants are trained to make rapid go responses toward certain items, and do not respond to other items. After the training, they tend to prefer go items over no-go items, and such preference change appears to be relatively long-lasting (Salomon et al., 2018; Schonberg et al., 2014). The current theoretical account for CAT primarily focuses on how selective attention toward go items may change people’s subsequent preference for these items (Itzkovitch et al., 2022; Schonberg & Katz, 2020). While selective attention processes may indeed play a role in the CAT effect, the current findings raise the question of whether part of the CAT effect can also be explained by action interpretation. Like in the GNG, participants may naturally interpret the go responses in the CAT as ‘take’ something, and no-go as ‘not take’ something (Houben, 2023), thereby assigning evaluative meaning to these responses. Rapid go responses may carry more positive connotation than slow go responses, because rapidly taking something is a clearer signal that the taken item is strongly preferred - akin to how people use shorter decision times to infer stronger preferences for the chosen item for other people (Gates et al., 2021). This may explain why go responses more effectively increase the evaluation of (Chen et al., 2016) and preference for go items (Schonberg et al., 2014) when they are executed rapidly. Examining the role of action interpretation in the CAT effect may allow us to develop more general theories of how actions influence evaluation (based on the observed commonalities), and highlight processes that may be specific to each of the training tasks (based on the observed differences). Such a more integrated theoretical understanding will also better guide the applied use of these interventions, in which the effective components can be more efficiently combined.

## CONCLUSION

Framing actions as go/no-go or approach/avoidance determined whether go/no-go or approach/avoidance actions influenced stimulus evaluation in a training that combined both, even though participants made the same motor responses. Features of executed go/no-go or approach/avoidance responses alone therefore do not seem sufficient to induce evaluative changes. Instead, the responses need to be interpreted as go/no-go or approach/avoidance actions to acquire their evaluative connotations. The role of action interpretation needs to be further examined, to yield useful theoretical and practical insights into how actions can best be utilized to change stimulus evaluation.

## FUNDING INFORMATION

Z.C. is supported by a Postdoctoral fellowship of the Scientific Research Foundation, Flanders (FWO-Vlaanderen; Grant Number 12A2322N). P.V.D. is support by grant BOF/STA/202202/004 of Ghent University.

## AUTHOR CONTRIBUTIONS

Z.C.: Conceptualization; Data curation; Formal analysis; Investigation; Methodology; Project administration; Software; Validation; Visualization; Writing – original draft; Writing – review & editing. P.V.D.: Conceptualization; Methodology; Writing – review & editing.

## DATA AVAILABILITY STATEMENT

All data, analysis code, research materials, and the Supplemental Materials are available at https://osf.io/ezgqw.

## Notes

^1^ In the experiments, we asked participants to report their “gender”, and provided four options for them to choose from, namely “female”, “male”, “non-binary”, and “I don’t want to say”. However, this question mixed up gender and sex. Sex refers to “the different biological and physiological characteristics of males and females”, while gender refers to “the socially constructed characteristics of women and men”, but can also go beyond these two categories (such as the “non-binary” option). To report the experimental procedure accurately and truthfully, we therefore used the label of “gender" here, and later on used the labels of “male” and “female” when reporting participant characteristics. However, we acknowledge that this is a mistake that we will rectify in our future research.^2^ In the pre-registrations of both experiments, we wrote “the training task immediately following training” for this exclusion criterion. However, this was a typo. There was no training task after training. It should be “the rating task immediately following training” instead.^3^ Note that in the pre-registration we only mentioned *BayesFactor*, but not JASP. Since JASP is largely based on *BayesFactor*, we used JASP to conduct Bayesian repeated-measures ANOVA, and Bayes factor robustness checks for paired-samples *t* tests.

## Supplementary Material


