# Supplementary material for: Action Interpretation Determines the Effects of Go/No-Go and Approach/Avoidance Actions on Stimulus Evaluation
Source: Open Mind (Camb). 2024 Jul 19;8:898–923. doi: 10.1162/opmi_a_00151 (PMC11285421; doi:10.1162/opmi_a_00151)
Supplement: Supplementary file 1 [file opmi-08-898-s001.pdf]

# Supplemental Materials for *Action interpretation determines the effects of go/no-go and approach/avoidance actions on stimulus evaluation*

Zhang Chen, Pieter Van Dessel

## Contents

**1 Comparing participant characteristics between experiments** **2**

**2 Performance in the training over time** **2**

2.1 Performance over time in Experiment 1 . . . . . . 2

2.2 Performance over time in Experiment 2 . . . . . . 3

**3 Effects of cue colors on training performance and stimulus evaluation** **4**

3.1 Effects of cue colors on training performance - Experiment 1 . . . . . . 4

3.2 Effects of cue colors on stimulus evaluation - Experiment 1 . . . . . . 5

3.3 Effects of cue colors on training performance - Experiment 2 . . . . . . 7

3.4 Effects of cue colors on stimulus evaluation - Experiment 2 . . . . . . 8

3.5 Summary and discussion . . . . . . 10

**4 Performance in the memory tasks** **11**

**5 Correlations between memory and training effects** **12**

5.1 Correlations between memory and training effects . . . . . . 13

5.2 Further exploratory analysis on memory . . . . . . 17

5.3 Summary and discussion . . . . . . 17

**6 Ratings after the memory tasks** **18**

# 1 Comparing participant characteristics between experiments

Table 1: Comparing participant characteristics between experiments

| Variable | Experiment 1 | Experiment 2 | Comparison |
|---|---|---|---|
| Age | 18.81 ($SD = 1.84$) | 18.84 ($SD = 2.36$) | $t(294.22) = -0.153$, $p = .879$ |
| Gender | 124 female, 23 male, 1 non-binary | 138 female, 19 male, 1 did not report gender | $\chi^2(1) = 0.531$, $p = .466^a$ |
| Hunger | 4.71 ($SD = 2.33$) | 4.74 ($SD = 2.55$) | $t(300.94) = -0.095$, $p = .925$ |
| Time since last meal[b] | 1.24 ($SD = 0.91$) | 1.32 ($SD = 0.92$) | $t(299.82) = -0.808$, $p = .420$ |
| Body mass index | 21.95 ($SD = 3.47$) | 21.77 ($SD = 3.47$) | $t(298.63) = 0.457$, $p = .648$ |
| Restraint eating score | 14.92 ($SD = 5.75$) | 14.39 ($SD = 6.43$) | $t(302.54) = 0.764$, $p = .446$ |
| Restraint eater status | 79 restrained, 68 non-restrained | 70 restrained, 88 non-restrained | $\chi^2(1) = 2.35$, $p = .125$ |

*Note.* a: For this comparison, the participant who indicated 'non-binary' in Experiment 1 and the one who did not report their gender in Experiment 2 were not included. b: 1 = *Less than 1 hour ago*, 2 = *1-3 hours ago*, 3 = *3-5 hours ago*, 4 = *More than 5 hours ago*.

# 2 Performance in the training over time

In the training, participants received 14 blocks in total, and received performance feedback after every two blocks and could take a short break if needed. Here we explored how performance in the different conditions might change over time.

## 2.1 Performance over time in Experiment 1

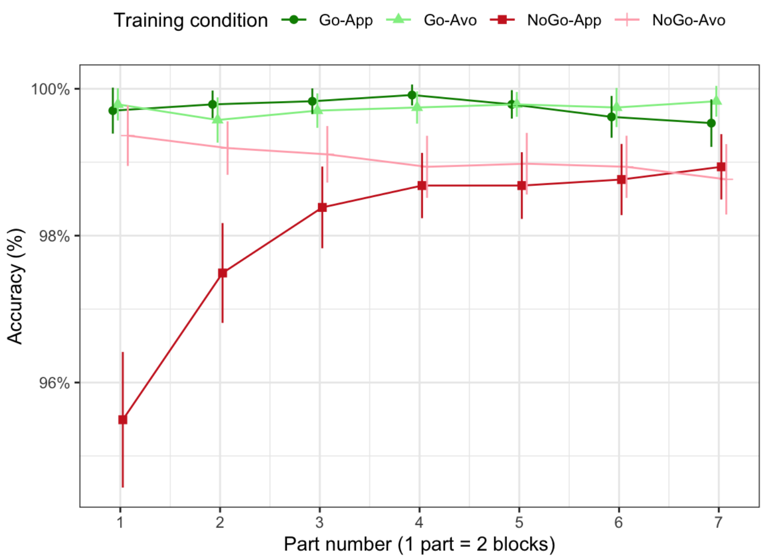


Figure 1: Accuracy in the training task over time in Experiment 1. The error bars stand for within-subject 95% confidence intervals. App = Approach, Avo = Avoidance.

For Experiment 1, we observed that the difference in accuracy between the no-go-avoidance and no-go-approach conditions was most pronounced in the first half of the training (from part 1 to part 3; Figure 1), and diminished in the

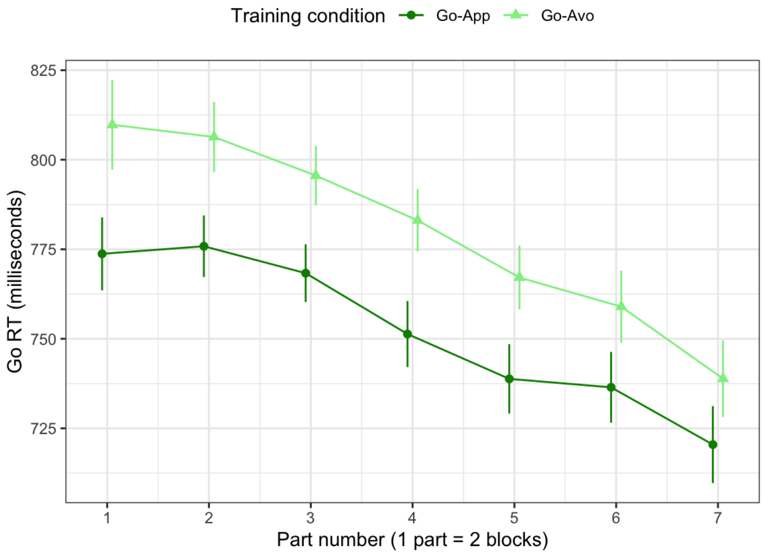


Figure 2: RTs on correct go trials in the training task over time in Experiment 1. The error bars stand for within-subject 95% confidence intervals. App = Approach, Avo = Avoidance.

second half of the training. The difference in go RT between the go-approach and go-avoidance conditions was visible throughout the whole training (Figure 2).

## 2.2 Performance over time in Experiment 2

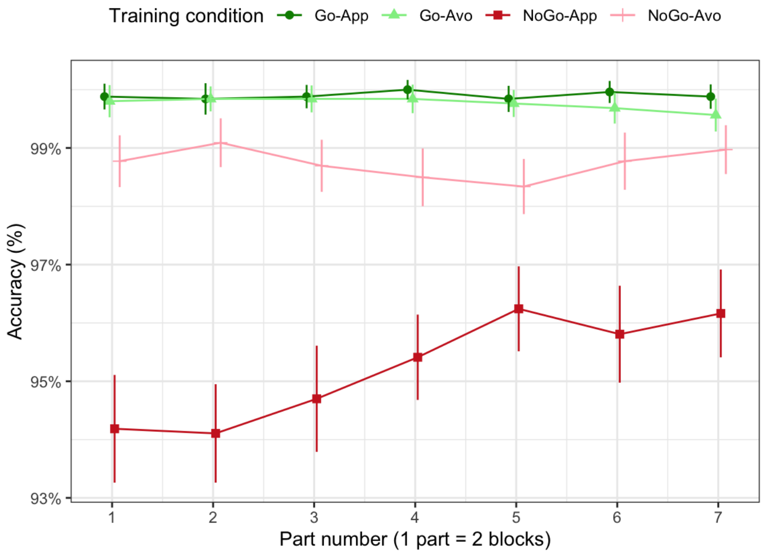


Figure 3: Accuracy in the training task over time in Experiment 2. The error bars stand for within-subject 95% confidence intervals. App = Approach, Avo = Avoidance.

For Experiment 2, we observed that the difference in accuracy between the no-go-avoidance and no-go-approach conditions was substantial and visible throughout the whole training (Figure 3). The difference in accuracy between the go-approach and go-avoidance conditions seemed to emerge especially in the later parts of the training. Note that there

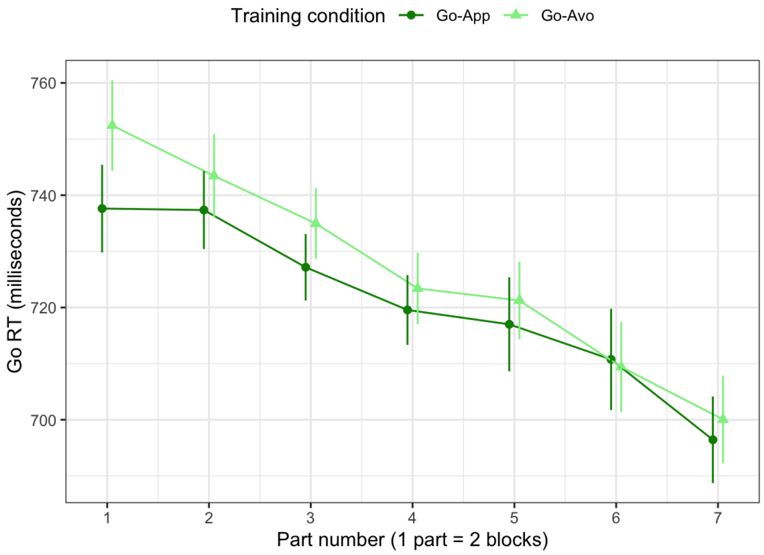


Figure 4: RTs on correct go trials in the training task over time in Experiment 2. The error bars stand for within-subject 95% confidence intervals. App = Approach, Avo = Avoidance.

was a difference in go RT between the go-approach and go-avoidance conditions in the earlier parts of the training, but diminished later on (Figure 4). That the performance difference between the go-approach and go-avoidance conditions emerged on either go accuracy or go RT in different parts of the training might be explained by a speed-accuracy trade-off.

# 3 Effects of cue colors on training performance and stimulus evaluation

In the training, we used blue and green frames around images to indicate the go/no-go (Experiment 1) or approach/avoidance conditions (Experiment 2). The assignment of colors into different training conditions was counterbalanced across participants, based on the order in which they opened the online study link. Note that this counterbalancing was not exact, as some participants might decide to not do the experiment after opening the link, and some might open the study link multiple times. Here, we explored whether cue colors influenced participants' performance in the training, and the training effects on stimulus evaluation.

## 3.1 Effects of cue colors on training performance - Experiment 1

In Experiment 1, for 78 participants, blue indicated 'go', green indicated 'no-go', while for the remaining 70 participants, green indicated 'go', blue indicated 'no-go'. To examine whether cue colors influenced participants' performance in the training in Experiment 1, we repeated the analysis conducted in the main text, with cue counterbalance as an extra between-subjects factor. Figure 5 shows the accuracies in the four training conditions, for the two counterbalance groups separately. Repeated-measures ANOVA shows none of the effects involving cue counterbalance is statistically significant (see Table 2), suggesting that cue colors did not influence accuracy in the training. Figure 6 shows the mean go response times in the go-approach and go-avoidance conditions, again for the two counterbalance groups separately. Here we observed a main effect of cue counterbalance (see Table 2). Perhaps contrary to what one may expect, participants made go responses

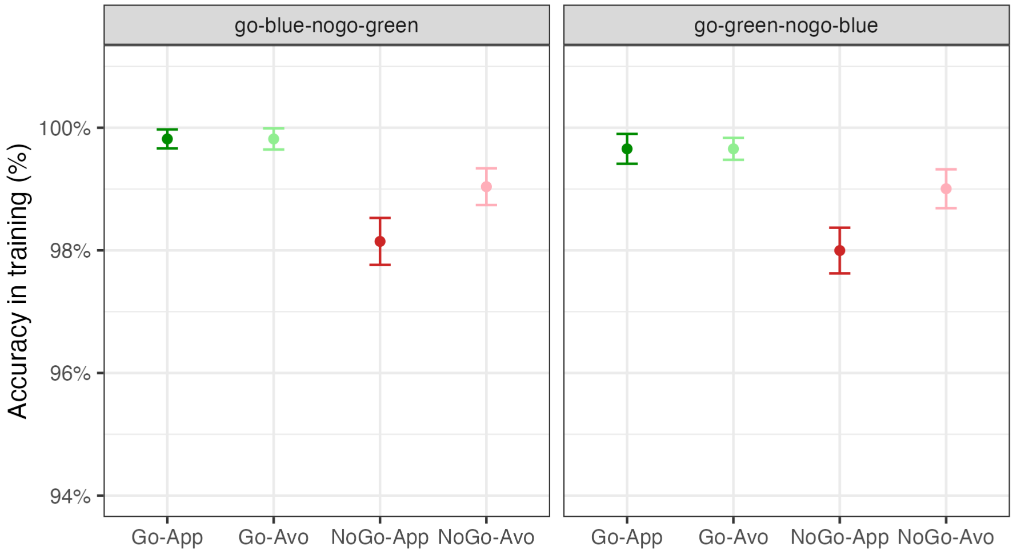


Figure 5: Accuracy in the training in Experiment 1, for the two cue counterbalance groups separately. The error bars stand for within-subject 95% confidence intervals. App = Approach, Avo = Avoidance.

Table 2: Results of ANOVAs on the effects of cue colors on training performance in Experiment 1.

| Effect | *df* | $MSE$ | $F$ | $ges$ | $p$ | $BF_{10}$ |
|---|---|---|---|---|---|---|
| *Accuracy in the training* | | | | | | |
| Motor Response (Go vs. NoGo) | 1, 146 | 0.021 | 156.45 | 0.180 | <.001 | $2.423 \times 10^{20}$ |
| Consequence (Approach vs. Avoidance) | 1, 146 | 0.003 | 23.16 | 0.034 | <.001 | 1080 |
| Cue Counterbalance | 1, 146 | $2.347 \times 10^{-4}$ | 1.058 | 0.002 | .305 | 0.186 |
| Motor Response * Consequence | 1, 146 | 0.003 | 21.68 | 0.034 | <.001 | $6.35 \times 10^{4}$ |
| Motor Response * Cue Counterbalance | 1, 146 | $1.815 \times 10^{-5}$ | 0.136 | $1.903 \times 10^{-4}$ | .713 | 0.161 |
| Consequence * Cue Counterbalance | 1, 146 | $1.230 \times 10^{-5}$ | 0.085 | $1.290 \times 10^{-4}$ | .770 | 0.157 |
| Motor Response * Consequence * Cue Counterbalance | 1, 146 | $1.225 \times 10^{-5}$ | 0.080 | $1.285 \times 10^{-4}$ | .778 | 0.183 |
| *Go RT in the training* | | | | | | |
| Consequence (Approach vs. Avoidance) | 1, 146 | $7.72 \times 10^{5}$ | 68.22 | 0.015 | <.001 | $6.366 \times 10^{10}$ |
| Cue Counterbalance | 1, 146 | $2.20 \times 10^{6}$ | 6.643 | 0.042 | .011 | 3.552 |
| Consequence * Cue Counterbalance | 1, 146 | 554.4 | 0.490 | $1.11 \times 10^{-4}$ | .485 | 0.226 |

more slowly when green indicated ‘go’, compared to the group for whom blue indicated ‘go’. Importantly, the interaction effect between consequence and cue counterbalance on go RT is not statistically significant, suggesting that approach and avoidance consequences affected go RT similarly when either green or blue indicated go responses.

## 3.2 Effects of cue colors on stimulus evaluation - Experiment 1

We then examined whether cue colors influenced the effect of the training on stimulus evaluation in Experiment 1 (Figure 7). To do this, we first conducted a repeated-measures ANOVA on the four trained conditions, with motor response (Go vs.

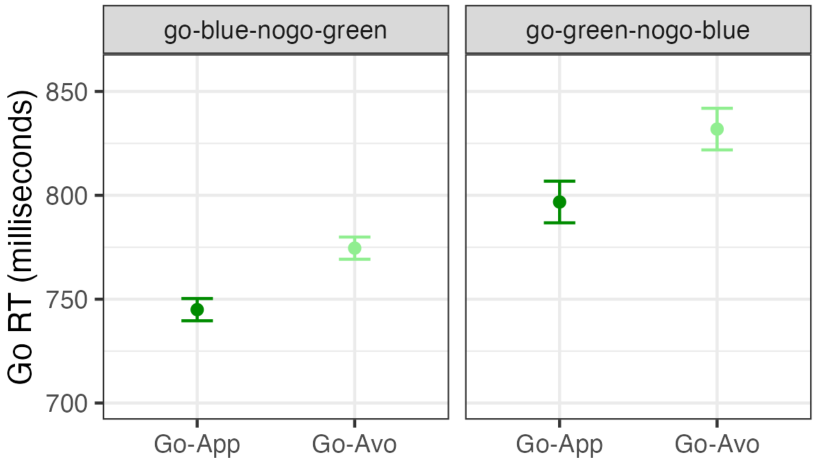


Figure 6: Go response times (in milliseconds) in the training task in Experiment 1, for the two cue-counterbalance groups separately. The error bars stand for within-subject 95% confidence intervals. App = Approach, Avo = Avoidance.

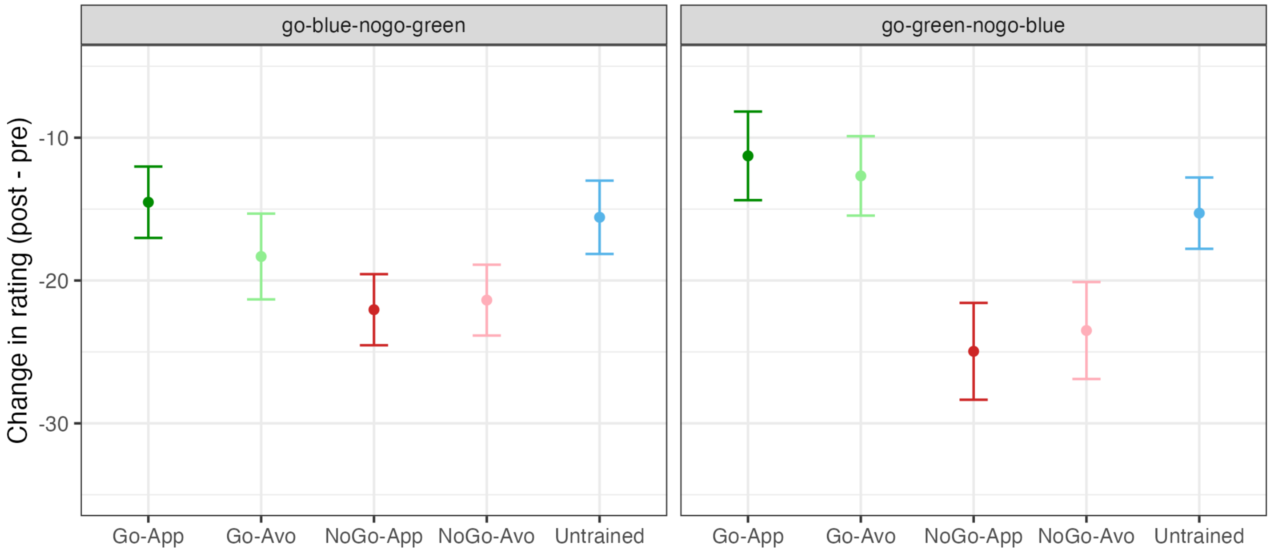


Figure 7: Changes in rating from before the training to immediately after the training in Experiment 1, for the two cue counterbalance groups separately. The error bars stand for within-subject 95% confidence intervals. App = Approach, Avo = Avoidance.

No-Go) and consequence (Approach vs. Avoidance) as within-subjects factors, cue counterbalance as a between-subjects factor, and changes in rating as the dependent variable.

The results of this ANOVA can be found in Table 3. Interestingly, we observed an interaction effect between motor response and cue counterbalance, such that the difference between go and no-go items was larger when green indicated ‘go’ and blue indicated ‘no-go’ than the other away around. This pattern is in line with the idea that green generally indicates ‘go’ or permission, and thus may have more positive connotation than blue.

To further examine whether the no-go devaluation effect was present in both groups, we conducted paired-samples t

Table 3: Results of ANOVA on the effects of cue colors on stimulus evaluation in Experiment 1.

| Effect | *df* | $MSE$ | $F$ | $ges$ | $p$ | $BF_{10}$ |
|---|---|---|---|---|---|---|
| Motor Response (Go vs. NoGo) | 1, 146 | 11354.08 | 51.26 | 0.057 | <.001 | $5.101 \times 10^7$ |
| Consequence (Approach vs. Avoidance) | 1, 146 | 87.13 | 0.584 | $4.653 \times 10^{-4}$ | .446 | 0.152 |
| Cue Counterbalance | 1, 146 | 136.60 | 0.169 | $7.293 \times 10^{-4}$ | .682 | 0.274 |
| Motor Response * Consequence | 1, 146 | 495.46 | 4.818 | 0.003 | .030 | 1.424 |
| Motor Response * Cue Counterbalance | 1, 146 | 1790.01 | 8.081 | 0.009 | .005 | 6.703 |
| Consequence * Cue Counterbalance | 1, 146 | 93.52 | 0.627 | $4.994 \times 10^{-4}$ | .430 | 0.192 |
| Motor Response * Consequence * Cue Counterbalance | 1, 146 | 23.95 | 0.233 | $1.280 \times 10^{-4}$ | .630 | 0.244 |

Table 4: Pairwise comparisons on changes in ratings (immediately after training) in Experiment 1, for the two cue counterbalance groups separately.

| Comparison | diff | lowerCI | upperCI | *df* | $t$ | $p$ | $BF_{10}$ | $d_z$ | $g_{av}$ |
|---|---|---|---|---|---|---|---|---|---|
| *Cue Group: Blue = Go, Green = NoGo* | | | | | | | | | |
| Go vs. NoGo | 5.29 | 2.74 | 7.84 | 77 | 4.13 | .001 | 220.4 | 0.468 | 0.326 |
| Go vs. Untrained | -0.85 | -4.05 | 2.36 | 77 | -0.52 | 1.000 | 0.142 | 0.059 | 0.060 |
| Untrained vs. NoGo | 6.13 | 3.06 | 9.21 | 77 | 3.98 | .001 | 132.9 | 0.450 | 0.431 |
| *Cue Group: Green = Go, Blue = NoGo* | | | | | | | | | |
| Go vs. NoGo | 12.25 | 7.95 | 16.56 | 69 | 5.68 | <.001 | $4.75 \times 10^4$ | 0.678 | 0.779 |
| Go vs. Untrained | 3.31 | 0.25 | 6.37 | 69 | 2.16 | .207 | 1.15 | 0.258 | 0.272 |
| Untrained vs. NoGo | 8.94 | 5.01 | 12.88 | 69 | 4.54 | <.001 | 790.8 | 0.542 | 0.591 |

*Note.* P values were corrected for multiple comparisons using the Bonferroni method.

tests to examine the main effects of go/no-go actions. Since approach vs. avoidance consequence had no effect on stimulus evaluation in Experiment 1, we combined the go-approach and go-avoidance conditions into the go condition, and the nogo-approach and nogo-avoidance conditions into the no-go condition. Go, no-go and untrained conditions were then compared with each other, for the two cue counterbalance groups separately. Table 4 shows the results. In both groups, we observed a no-go devaluation effect: no-go items were evaluated less positively than both go and untrained items. While cue colors moderated the effects of go/no-go actions on stimulus evaluation, the changes in ratings cannot be explained by cue colors alone, as the effects were also present in the go-blue-nogo-green group.

## 3.3 Effects of cue colors on training performance - Experiment 2

In Experiment 2, for 72 participants blue indicated 'approach' and green indicated 'avoidance', and for 86 participants green indicated 'approach' and blue indicated 'avoidance'. For Experiment 2, we observed a statistically significant main effect of cue counterbalance group on accuracy (Table 5). When green indicated 'approach' and blue indicated 'avoidance', participants were overall slightly more accurate than when blue indicated 'approach' and green indicated 'avoidance' (Figure 8). The direction of this effect is in line with the idea that green generally indicates permission, and may therefore

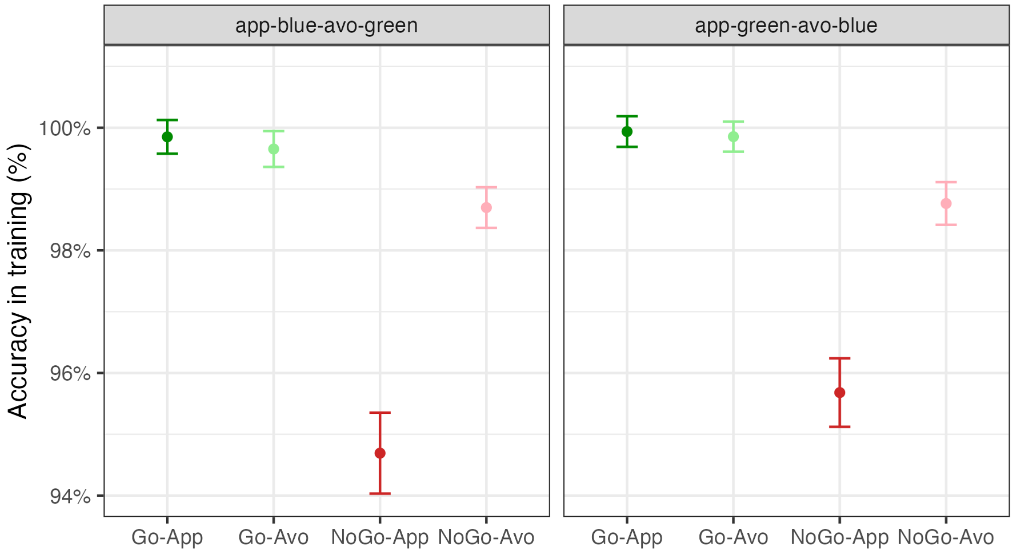


Figure 8: Accuracy in the training in Experiment 2, for the two cue-counterbalance groups separately. The error bars stand for within-subject 95% confidence intervals. App = Approach, Avo = Avoidance.

Table 5: Results of ANOVAs on the effects of cue colors on training performance in Experiment 2.

| Effect | *df* | $MSE$ | $F$ | $ges$ | $p$ | $BF_{10}$ |
|---|---|---|---|---|---|---|
| *Accuracy in the training* | | | | | | |
| Motor Response (Go vs. NoGo) | 1, 156 | 0.129 | 337.41 | 0.379 | <.001 | $1.057 \times 10^{36}$ |
| Consequence (Approach vs. Avoidance) | 1, 156 | 0.045 | 169.43 | 0.177 | <.001 | $1.431 \times 10^{18}$ |
| Cue Counterbalance | 1, 156 | 0.002 | 4.169 | 0.008 | .043 | 0.831 |
| Motor Response * Consequence | 1, 156 | 0.053 | 189.37 | 0.201 | <.001 | $2.057 \times 10^{36}$ |
| Motor Response * Cue Counterbalance | 1, 156 | $5.744 \times 10^{-4}$ | 1.506 | 0.003 | .222 | 0.281 |
| Consequence * Cue Counterbalance | 1, 156 | $6.363 \times 10^{-4}$ | 2.374 | 0.003 | .125 | 0.332 |
| Motor Response * Consequence * Cue Counterbalance | 1, 156 | 0.001 | 3.745 | 0.005 | .055 | 1.303 |
| *Go RT in the training* | | | | | | |
| Consequence (Approach vs. Avoidance) | 1, 156 | 387.23 | 0.503 | $2.014 \times 10^{-4}$ | .479 | 0.152 |
| Cue Counterbalance | 1, 156 | 9184.91 | 0.795 | 0.005 | .374 | 0.502 |
| Consequence * Cue Counterbalance | 1, 156 | 835.39 | 1.086 | $4.343 \times 10^{-4}$ | .299 | 0.283 |

facilitate approach actions. However, the size of this effect appeared to be rather small, and the Bayes factor revealed inconclusive evidence. For go response times, we observed no effect of the cue counterbalance group (Table 5 and Figure 9).

## 3.4 Effects of cue colors on stimulus evaluation - Experiment 2

We similarly examined the potential influence of cue colors on the training effect (Figure 10), by submitting the four trained conditions to a repeated-measures ANOVA. Motor response (Go vs. NoGo) and consequence (Approach vs. Avoidance) were included as within-subjects factors, and cue counterbalance was included as a between-subjects factor. All effects

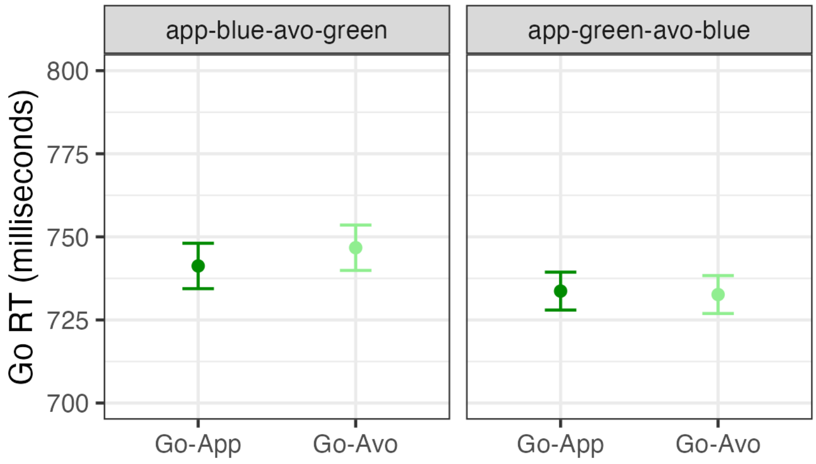


Figure 9: Go response times (in milliseconds) in the training task in Experiment 2, for the two cue-counterbalance groups separately. The error bars stand for within-subject 95% confidence intervals. App = Approach, Avo = Avoidance.

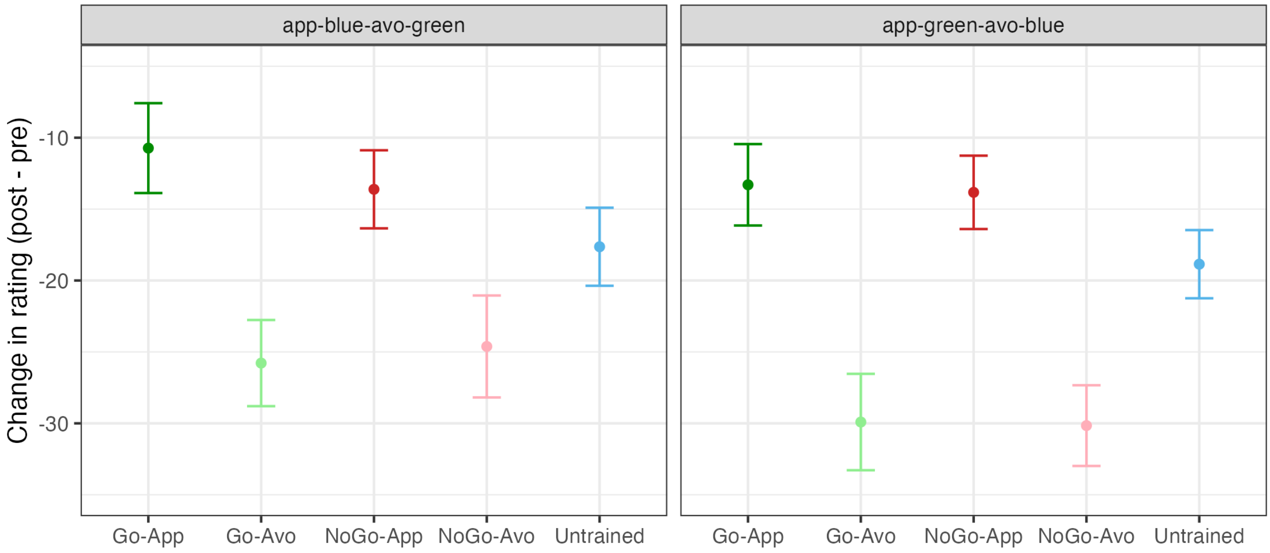


Figure 10: Changes in rating from before the training to immediately after the training in Experiment 2, for the two cue-counterbalance groups separately. The error bars stand for within-subject 95% confidence intervals. App = Approach, Avo = Avoidance.

involving cue counterbalance were not statistically significant (Table 6). For the sake of consistency with Experiment 1, we nevertheless still examined the main effects of approach/avoidance actions for the two cue groups separately. For this analysis, we combined the go-approach and nogo-approach conditions into the approach condition, and the go-avoidance and nogo-avoidance conditions into the avoidance condition. Approach, avoidance and untrained conditions were then compared with each other using paired-samples t tests (Table 7). In both groups, we observed that approached items were evaluated more positively than untrained items, which in turn were evaluated more positively than avoided items.

Table 6: Results of ANOVA on the effects of cue colors on stimulus evaluation in Experiment 2.

| Effect | $df$ | $MSE$ | $F$ | $ges$ | $p$ | $BF_{10}$ |
|---|---|---|---|---|---|---|
| Motor Response (Go vs. NoGo) | 1, 156 | 61.30 | 0.590 | $2.513 \times 10^{-4}$ | .444 | 0.121 |
| Consequence (Approach vs. Avoidance) | 1, 156 | 34102.00 | 106.8 | 0.123 | <.001 | $5.979 \times 10^{16}$ |
| Cue Counterbalance | 1, 156 | 1518.34 | 1.508 | 0.006 | .221 | 0.498 |
| Motor Response * Consequence | 1, 156 | 183.50 | 1.383 | $7.519 \times 10^{-4}$ | .241 | 0.244 |
| Motor Response * Cue Counterbalance | 1, 156 | 8.72 | 0.084 | $3.574 \times 10^{-5}$ | .773 | 0.149 |
| Consequence * Cue Counterbalance | 1, 156 | 464.61 | 1.455 | 0.002 | .230 | 0.373 |
| Motor Response * Consequence * Cue Counterbalance | 1, 156 | 139.22 | 1.049 | $5.706 \times 10^{-4}$ | .307 | 0.355 |

Table 7: Pairwise comparisons on changes in ratings (immediately after training) in Experiment 2, for the two cue counterbalance groups separately.

| Comparison | diff | lowerCI | upperCI | $df$ | $t$ | $p$ | $BF_{10}$ | $d_z$ | $g_{av}$ |
|---|---|---|---|---|---|---|---|---|---|
| *Cue Group: Blue = Approach, Green = Avoidance* | | | | | | | | | |
| Approach vs. Avoidance | 13.03 | 8.61 | 17.45 | 71 | 5.88 | <.001 | $1.08 \times 10^{5}$ | 0.693 | 0.717 |
| Approach vs. Untrained | 5.46 | 2.23 | 8.69 | 71 | 3.37 | .007 | 21.25 | 0.398 | 0.348 |
| Untrained vs. Avoidance | 7.56 | 3.32 | 11.81 | 71 | 3.55 | .004 | 35.69 | 0.419 | 0.385 |
| *Cue Group: Green = Approach, Blue = Avoidance* | | | | | | | | | |
| Approach vs. Avoidance | 16.47 | 12.82 | 20.12 | 85 | 8.96 | <.001 | $1.15 \times 10^{11}$ | 0.967 | 0.925 |
| Approach vs. Untrained | 5.29 | 2.41 | 8.18 | 85 | 3.65 | .003 | 48.25 | 0.393 | 0.329 |
| Untrained vs. Avoidance | 11.18 | 7.63 | 14.73 | 85 | 6.26 | <.001 | $7.75 \times 10^{5}$ | 0.675 | 0.586 |

*Note.* P values were corrected for multiple comparisons using the Bonferroni method.

## 3.5 Summary and discussion

Overall, cue colors did not strongly influence participants' performance in the training in both experiments. In Experiment 1, we observed that participants made go responses more *slowly* when 'go' was indicated by green compared to when 'go' was indicated by blue. This pattern is opposite to what one may expect based on the idea that green may facilitate go responses as it is often used as a go signal. Cue colors did not influence participants' accuracy in the training in Experiment 1. In Experiment 2 where the cues indicated the approach/avoidance conditions, participants overall were more accurate when 'approach' was indicated by green compared to when 'approach' was indicated by blue. While this pattern is in line with the idea that green may facilitate approach actions, the size of this effect is rather small (the Bayes factor revealed inconclusive evidence for this effect). No effect of cue colors was observed on the go response times in Experiment 2. Overall, we conclude that the influence of cue colors on participants' performance in the training is very limited.

For stimulus evaluation, we observed different patterns in the two experiments. In Experiment 1, we observed that the difference in changes in rating between go and no-go items was larger when green indicated 'go' and blue indicated 'no-go', compared to when blue indicated 'go' and green indicated 'no-go'. In Experiment 2, cue colors did not significantly influence the effects of approach/avoidance actions on stimulus evaluation. Importantly, further exploratory analyses in the

two cue counterbalance groups separately showed an effect of go/no-go actions on stimulus evaluation in both groups in Experiment 1. Similarly, an effect of approach/avoidance actions on stimulus evaluation was also observed in both groups in Experiment 2. These results suggest that the observed effects of training on stimulus evaluation cannot be explained by the cue colors alone, as the effects also emerged when green indicated 'no-go' or 'avoidance' and blue indicated 'go' or 'approach'.

We speculate that the approach and avoidance consequences in the training (i.e., food items falling inside or outside one's shopping cart) may be interpreted unambiguously and thus carry clear evaluative connotations. This may be the reason why cue colors did not influence the effects of approach/avoidance actions on stimulus evaluation in Experiment 2. In contrast, the go/no-go actions in Experiment 1 may be less clearly interpreted. Using green to indicate 'go' responses and blue to indicate 'no-go' responses may have helped participants to interpret making 'go' responses as 'taking something' and making 'no-go' responses as 'not taking something' (e.g., Houben, 2023), which may explain why the 'green = go, blue = no-go' group showed a slightly larger effect of go/no-go actions on stimulus evaluation than the 'blue = go, green = no-go' group.

# 4 Performance in the memory tasks

Immediately after the training, participants received two memory tasks to probe their memory for whether each item was paired with go or no-go responses, and with approach or avoidance consequences, respectively. Responses in the memory tasks were first coded. For the go/no-go memory task, we coded 'sure did not press' as -2, 'maybe did not press' as -1, 'do not remember' as 0, 'maybe pressed' as 1, and 'sure pressed' as 2. For the approach/avoidance memory task, we coded 'sure outside' as -2, 'maybe outside' as -1, 'do not remember' as 0, 'maybe inside' as 1, and 'sure inside' as 2.

For each memory task, we first computed the average response for each condition, for each participant separately. We then submitted the memory responses for the four trained conditions to a 2 (motor response, go vs. no-go) by 2 (consequence, approach vs. avoidance) repeated-measures ANOVA (both the frequentist and Bayesian versions). The results are shown in Table 8. In Experiment 1, where the cues were about go and no-go responses, participants overall remembered well whether each item was paired with go or no-go responses, as would be expected (Figure 11-A1). The approach/avoidance consequences did not influence participants' memory for go/no-go responses. They could to some extent also remember whether each item was paired with approach or avoidance (Figure 11-B1). Furthermore, participants were more likely to report approach consequence for go items compared to no-go items, which again might reflect the interaction between go/no-go responses and approach/avoidance consequences as observed in the training. In Experiment 2, where the cues were about approach/avoidance, participants overall remembered well whether each item was paired with approach or avoidance (Figure 11-B2). The go/no-go responses did not influence participants' memory for approach/avoidance consequences. Participants could to some extent also remember whether each item was paired with go or no-go responses (Figure 11-A2). We also observed an interaction effect for the go/no-go memory in Experiment 2, such that participants remembered the go/no-go condition better when the consequence was approach compared to avoidance.

We then conducted a series of pairwise comparisons for each memory task in each experiment. First, we compared the trained conditions, by either holding the consequence constant (i.e., Go-App vs. NoGo-App, and Go-Avo vs. NoGo-Avo) or the motor response constant (i.e., Go-App vs. Go-Avo, and NoGo-App vs. NoGo-Avo). We then compared each of the

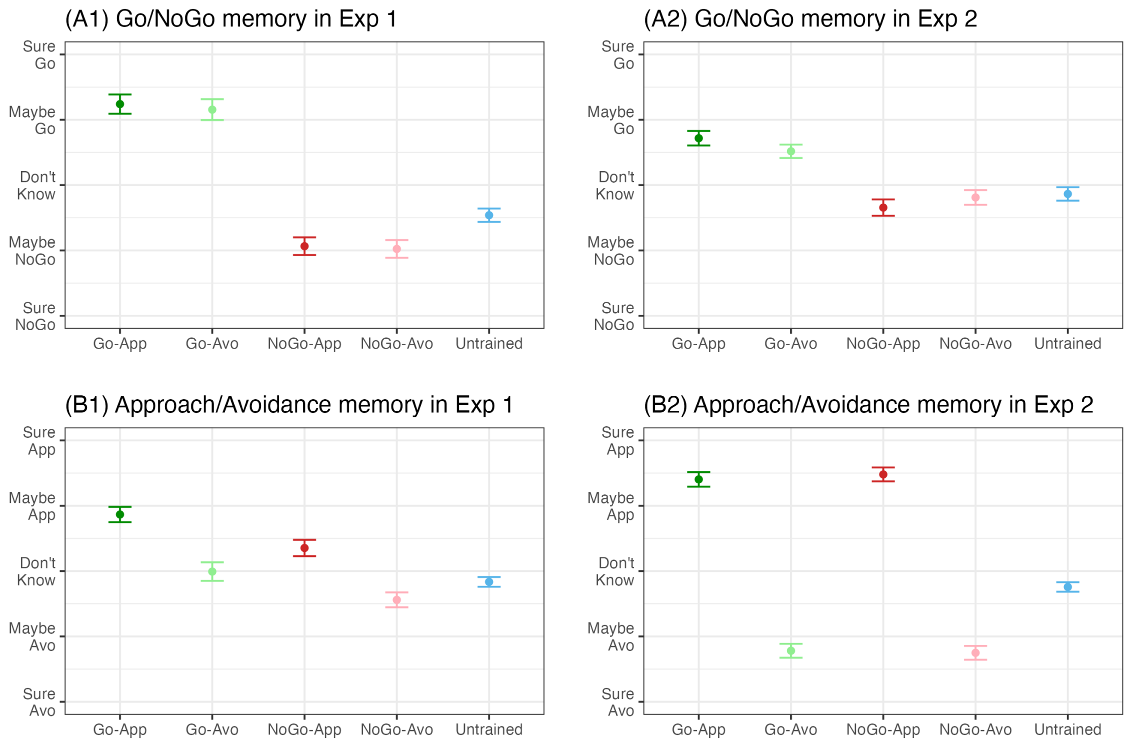


Figure 11: Memory performance in both tasks in Experiment 1 (left, A1 and B1) and Experiment 2 (right, A2 and B2). The error bars stand for within-subject 95% confidence intervals. App = Approach. Avo = Avoidance.

four trained conditions against the untrained condition. The p values of these 8 paired-samples t tests were corrected for multiple comparisons using the Bonferroni method. The results of all pairwise comparisons are shown in Table 9.

Lastly, we directly compared participants' memory performance between the two experiments. For the go/no-go memory task, we computed the difference in memory between the go (i.e., go-approach and go-avoidance) and no-go conditions (i.e., no-go-approach and no-go-avoidance) for each participant. A larger difference means participants could better distinguish between the go and no-go items in their memory. As would be expected, participants in Experiment 1 (where the cues were about go and no-go responses) showed a larger difference than those in Experiment 2, diff = 1.27, 95% CI = [0.99, 1.55], $t(240.4) = 8.93$, $p < .001$, $BF = 3.16 \times 10^{14}$, Hedge's g = 1.02. For the approach/avoidance memory task, we similarly computed the difference in memory between the approach (i.e., go-approach and no-go-approach) and avoidance conditions (i.e., go-avoidance and no-go-avoidance). Again, as would be expected, participants in Experiment 2 (where the cues were about approach and avoidance consequences) showed a larger difference than those in Experiment 1, diff = 1.84, 95% CI = [1.60, 2.09], $t(303.0) = 14.82$, $p < .001$, $BF = 1.38 \times 10^{34}$, Hedge's g = 1.69.

# 5 Correlations between memory and training effects

Some previous work has shown that the effects of GNG (Chen & Veling, 2022; Liu et al., 2023) and AAT (Van Dessel et al., 2016) were related to participants' memory of stimulus-action relation. We therefore explored the role of stimulus-action memory in the training effects in our novel task.

Table 8: Results of ANOVAs on performance in the memory tasks in Experiments 1 and 2.

| Effect | *df* | $MSE$ | $F$ | $ges$ | $p$ | $BF$ |
|---|---|---|---|---|---|---|
| *Go/NoGo memory in Experiment 1* | | | | | | |
| Go vs. NoGo | 1, 146 | 2.19 | 311.74 | .628 | <.001 | $1.718 \times 10^{125}$ |
| Approach vs. Avoidance | 1, 146 | 0.15 | 3.94 | .001 | .049 | 0.148 |
| Interaction | 1, 146 | 0.11 | 0.62 | <.001 | .433 | 0.119 |
| *Approach/Avoidance memory in Experiment 1* | | | | | | |
| Go vs. NoGo | 1, 146 | 0.52 | 62.96 | .093 | <.001 | $3.536 \times 10^{11}$ |
| Approach vs. Avoidance | 1, 146 | 1.11 | 92.16 | .243 | <.001 | $5.836 \times 10^{34}$ |
| Interaction | 1, 146 | 0.21 | 1.09 | <.001 | .298 | 0.163 |
| *Go/NoGo memory in Experiment 2* | | | | | | |
| Go vs. NoGo | 1, 157 | 0.84 | 147.05 | .253 | <.001 | $4.842 \times 10^{41}$ |
| Approach vs. Avoidance | 1, 157 | 0.51 | 0.16 | <.001 | .692 | 0.095 |
| Interaction | 1, 157 | 0.22 | 22.89 | .013 | <.001 | 12.807 |
| *Approach/Avoidance memory in Experiment 2* | | | | | | |
| Go vs. NoGo | 1, 157 | 0.11 | 0.74 | <.001 | .392 | 0.095 |
| Approach vs. Avoidance | 1, 157 | 1.25 | 905.06 | .817 | <.001 | $8.614 \times 10^{231}$ |
| Interaction | 1, 157 | 0.10 | 4.64 | .002 | .033 | 0.211 |

## 5.1 Correlations between memory and training effects

Responses in the memory tasks were first coded in the same way as above. For the go/no-go memory task, we coded 'sure did not press' as -2, 'maybe did not press' as -1, 'do not remember' as 0, 'maybe pressed' as 1, and 'sure pressed' as 2. For each participant, we then computed their average scores in the go/no-go memory task for go and no-go items separately. For instance, for a participant who correctly chose 'sure pressed' for all go items, their average memory score for go items would be 2. If they also correctly chose 'sure did not press' for all no-go items, their average memory score for no-go items would be -2. We then computed the difference between these two memory scores, which for this hypothetical participant would be 4. Note that 4 is the maximum score a participant could possibly get in the go/no-go memory task. In contrast, if a participant incorrectly selected 'sure did not press' for all go items (i.e., a memory score of -2) and 'sure pressed' for all no-go items (i.e., a memory score of 2), the difference in memory scores for go and no-go items would be -4. -4 is the minimum score a participant could possibly get. A positive GNG memory index (i.e., the difference score) thus indicated that participants were more likely to report 'go' for go items than no-go items, with a larger value standing for a better memory of the trained go/no-go contingencies.

For the approach/avoidance memory task, we coded 'sure outside' as -2, 'maybe outside' as -1, 'do not remember' as 0, 'maybe inside' as 1, and 'sure inside' as 2. For each participant, we similarly computed their average scores in the approach/avoidance memory task for the approach and avoidance items separately. We then used the difference between the approach and avoidance items as the AAT memory index. The AAT memory index is similarly bounded between 4

Table 9: Pairwise comparisons on performance in the memory tasks in Experiments 1 and 2.

| Comparison | diff | lowerCI | upperCI | *df* | $t$ | $p$ | $BF_{10}$ | $d_z$ | $g_{av}$ |
|---|---|---|---|---|---|---|---|---|---|
| *Comparisons on Go/NoGo memory in Experiment 1* | | | | | | | | | |
| Go-App vs. NoGo-App | 2.18 | 1.93 | 2.42 | 146 | 17.83 | <.001 | $1.44 \times 10^{35}$ | 1.471 | 2.643 |
| Go-Avo vs. NoGo-Avo | 2.13 | 1.88 | 2.38 | 146 | 16.65 | <.001 | $1.82 \times 10^{32}$ | 1.373 | 2.540 |
| Go-App vs. Go-Avo | 0.09 | -0.00 | 0.17 | 146 | 1.87 | .503 | 0.504 | 0.155 | 0.112 |
| NoGo-App vs. NoGo-Avo | 0.04 | -0.03 | 0.12 | 146 | 1.08 | 1.000 | 0.162 | 0.089 | 0.046 |
| Go-App vs. Untrained | 1.70 | 1.51 | 1.89 | 146 | 17.40 | <.001 | $1.25 \times 10^{34}$ | 1.435 | 2.304 |
| Go-Avo vs. Untrained | 1.62 | 1.41 | 1.82 | 146 | 15.78 | <.001 | $1.27 \times 10^{30}$ | 1.302 | 2.107 |
| NoGo-App vs. Untrained | -0.47 | -0.62 | -0.33 | 146 | -6.62 | <.001 | $1.38 \times 10^{7}$ | 0.546 | 0.573 |
| NoGo-Avo vs. Untrained | -0.52 | -0.65 | -0.38 | 146 | -7.36 | <.001 | $6.49 \times 10^{8}$ | 0.607 | 0.632 |
| *Comparisons on Approach/Avoidance memory in Experiment 1* | | | | | | | | | |
| Go-App vs. NoGo-App | 0.51 | 0.38 | 0.65 | 146 | 7.49 | <.001 | $1.28 \times 10^{9}$ | 0.618 | 0.744 |
| Go-Avo vs. NoGo-Avo | 0.43 | 0.29 | 0.58 | 146 | 5.94 | <.001 | $5.01 \times 10^{5}$ | 0.490 | 0.553 |
| Go-App vs. Go-Avo | 0.87 | 0.68 | 1.07 | 146 | 9.00 | <.001 | $5.36 \times 10^{12}$ | 0.742 | 1.128 |
| NoGo-App vs. NoGo-Avo | 0.79 | 0.61 | 0.98 | 146 | 8.58 | <.001 | $5.08 \times 10^{11}$ | 0.708 | 1.138 |
| Go-App vs. Untrained | 1.03 | 0.90 | 1.17 | 146 | 15.19 | <.001 | $4.11 \times 10^{28}$ | 1.253 | 1.942 |
| Go-Avo vs. Untrained | 0.16 | -0.01 | 0.32 | 146 | 1.89 | .481 | 0.523 | 0.156 | 0.250 |
| NoGo-App vs. Untrained | 0.52 | 0.39 | 0.65 | 146 | 8.08 | <.001 | $3.07 \times 10^{10}$ | 0.666 | 0.952 |
| NoGo-Avo vs. Untrained | -0.28 | -0.40 | -0.15 | 146 | -4.36 | <.001 | 597.6 | 0.360 | 0.511 |
| *Comparisons on Go/NoGo memory in Experiment 2* | | | | | | | | | |
| Go-App vs. NoGo-App | 1.06 | 0.89 | 1.23 | 157 | 12.36 | <.001 | $6.53 \times 10^{21}$ | 0.984 | 1.356 |
| Go-Avo vs. NoGo-Avo | 0.71 | 0.55 | 0.86 | 157 | 9.11 | <.001 | $1.61 \times 10^{13}$ | 0.725 | 0.964 |
| Go-App vs. Go-Avo | 0.20 | 0.07 | 0.33 | 157 | 3.15 | .016 | 10.02 | 0.251 | 0.300 |
| NoGo-App vs. NoGo-Avo | -0.16 | -0.30 | -0.01 | 157 | -2.15 | .263 | 0.837 | 0.171 | 0.183 |
| Go-App vs. Untrained | 0.85 | 0.71 | 1.00 | 157 | 11.65 | <.001 | $8.04 \times 10^{19}$ | 0.927 | 1.484 |
| Go-Avo vs. Untrained | 0.65 | 0.52 | 0.79 | 157 | 9.41 | <.001 | $9.65 \times 10^{13}$ | 0.749 | 1.164 |
| NoGo-App vs. Untrained | -0.21 | -0.38 | -0.04 | 157 | -2.42 | .133 | 1.50 | 0.193 | 0.309 |
| NoGo-Avo vs. Untrained | -0.05 | -0.20 | 0.10 | 157 | -0.71 | 1.000 | 0.113 | 0.056 | 0.084 |
| *Comparisons on Approach/Avoidance memory in Experiment 2* | | | | | | | | | |
| Go-App vs. NoGo-App | -0.08 | -0.14 | -0.01 | 157 | -2.26 | .199 | 1.06 | 0.180 | 0.131 |
| Go-Avo vs. NoGo-Avo | 0.03 | -0.04 | 0.11 | 157 | 0.83 | 1.000 | 0.124 | 0.066 | 0.046 |
| Go-App vs. Go-Avo | 2.62 | 2.44 | 2.81 | 157 | 28.20 | <.001 | $5.68 \times 10^{59}$ | 2.243 | 4.080 |
| NoGo-App vs. NoGo-Avo | 2.73 | 2.55 | 2.91 | 157 | 29.74 | <.001 | $6.16 \times 10^{62}$ | 2.366 | 4.371 |
| Go-App vs. Untrained | 1.65 | 1.51 | 1.78 | 157 | 24.06 | <.001 | $1.08 \times 10^{51}$ | 1.914 | 2.953 |
| Go-Avo vs. Untrained | -0.98 | -1.10 | -0.86 | 157 | -16.12 | <.001 | $6.53 \times 10^{31}$ | 1.282 | 1.624 |
| NoGo-App vs. Untrained | 1.72 | 1.59 | 1.85 | 157 | 26.50 | <.001 | $1.89 \times 10^{56}$ | 2.108 | 3.188 |
| NoGo-Avo vs. Untrained | -1.01 | -1.12 | -0.89 | 157 | -17.42 | <.001 | $1.56 \times 10^{35}$ | 1.386 | 1.679 |

*Note.* P values were corrected for multiple comparisons using the Bonferroni method for the four sets of analyses separately.

and -4, with a larger value standing for a better memory of the trained approach/avoidance contingencies.

We then examined the correlations between these memory indices and the corresponding training effects (Figure 12). In line with previous findings, we observed that on the participant level, those who remembered the GNG contingencies better in Experiment 1 showed a larger GNG effect, whereas those who remembered the AAT contingencies better in Experiment 2 showed a larger AAT effect. Interestingly, in Experiment 1 where the cues indicated go/no-go responses, we still observed a positive correlation between participants' AAT memory and the AAT effect (but see below, where

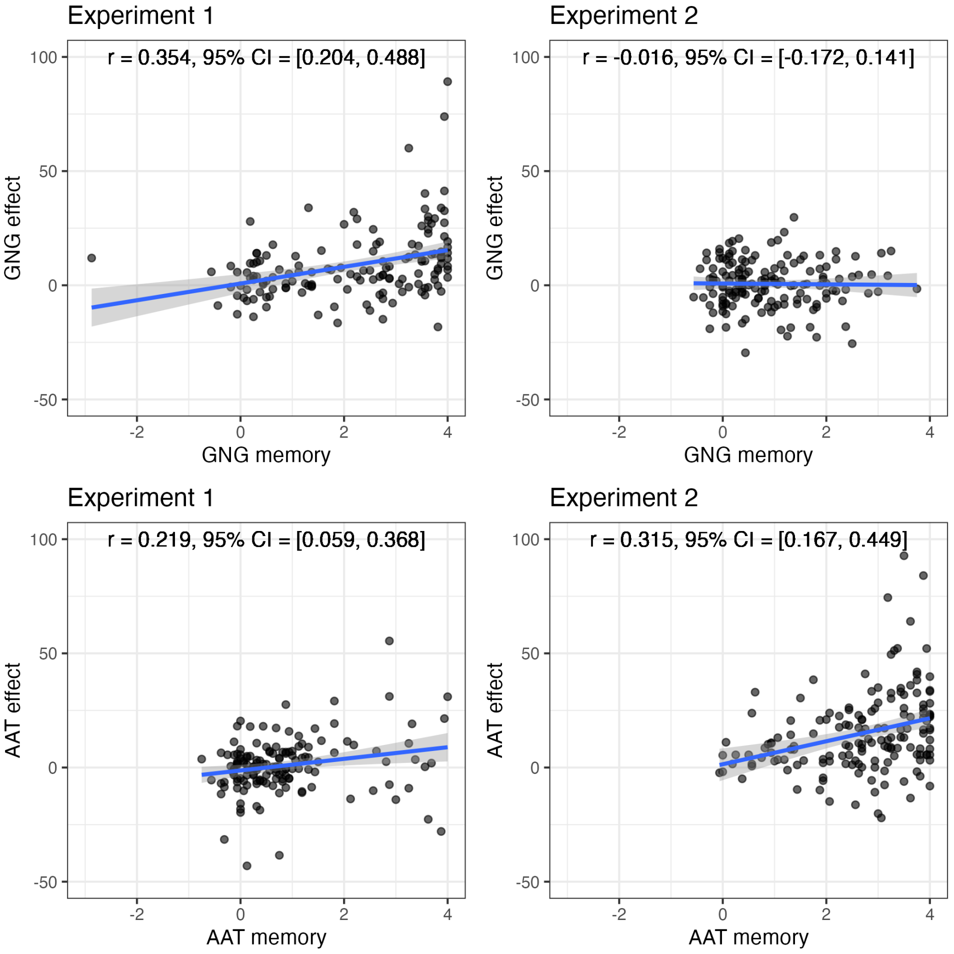


Figure 12: Correlations between stimulus-action memory and the corresponding training effect. r = Pearson's correlation coefficient, 95% CI = 95% confidence intervals.

this correlation was only statistically significant when the motor response was go, but not when the motor response was no-go). That is, although overall we did not observe an AAT effect in Experiment 1, those who remembered the AAT contingencies better showed a larger AAT effect. In contrast, in Experiment 2 where the cues indicated approach and avoidance actions, the correlation between participants' GNG memory and the GNG effect was not statistically significant. We further compared the correlations, using the R package *cocor* (version 1.1-4; Diedenhofen & Musch, 2015). The correlation between GNG memory and GNG effect was stronger in Experiment 1 than in Experiment 2, diff = 0.370, 95% CI = [0.153, 0.576], z = 3.33, $p < .001$, while the correlation between AAT memory and AAT effect was not statistically stonger in Experiment 2 than in Experiment 1, diff = 0.096, 95% CI = [-0.115, 0.305], z = 0.888, $p = .375$. We also compared the correlation for GNG in Experiment 1 and the correlation for AAT in Experiment 2 (i.e., the two cued dimensions), and found the difference to be not statistically significant, diff = 0.040, 95% CI = [-0.162, 0.240], z = 0.384, $p = .700$. In contrast, the difference between the correlation for AAT in Experiment 1 and that for GNG in Experiment 2 (i.e., the two uncued dimensions) was statistically significant, diff = 0.235, 95% CI = [0.010, 0.452], z = 2.05, $p = .040$.

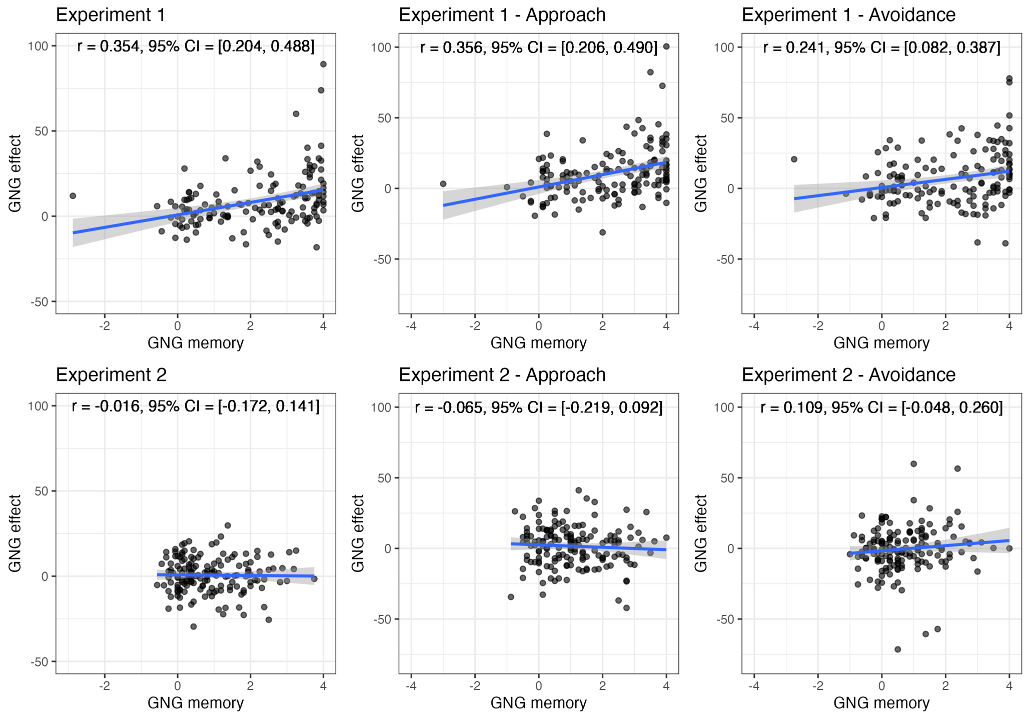


Figure 13: Correlations between GNG memory and GNG effect (left: overall; middle: when the consequence was approach; right: when the consequence was avoidance). r = Pearson's correlation coefficient, 95% CI = 95% confidence intervals.

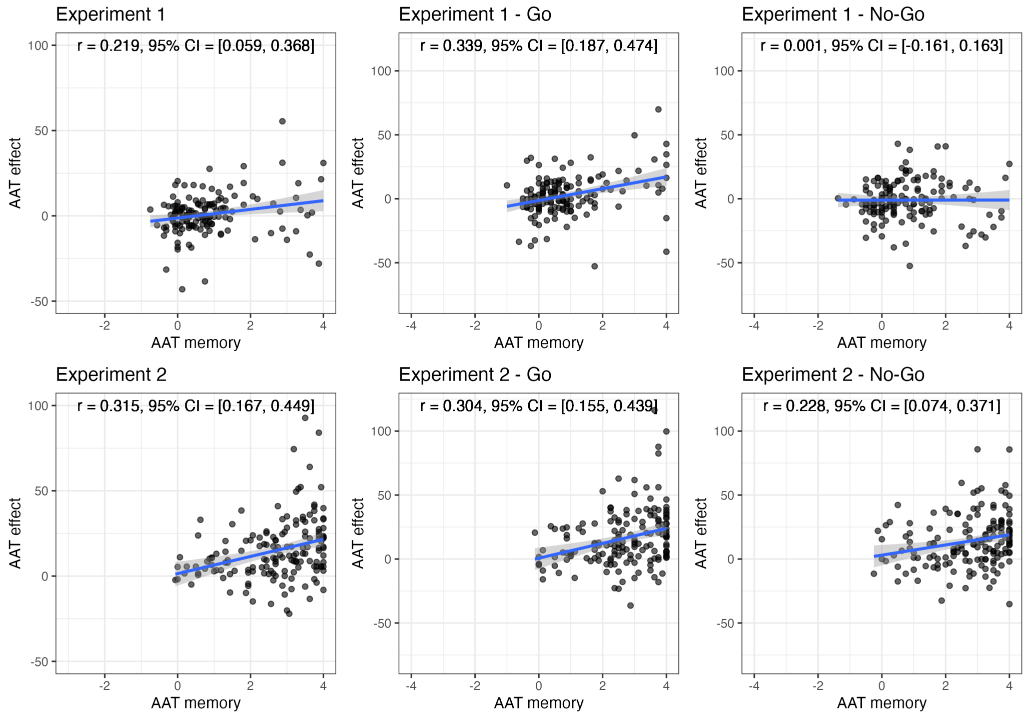


Figure 14: Correlations between AAT memory and AAT effect (left: overall; middle: when the response was go; right: when the response was no-go). r = Pearson's correlation coefficient, 95% CI = 95% confidence intervals.

## 5.2 Further exploratory analysis on memory

In the analysis above, we explored the correlations between stimulus-action memory and the corresponding training effect, by collapsing the two levels on the uncued dimension. Here, we further explored whether the same results would hold when we took the uncued action dimension into account. Figure 13 shows the correlations between GNG memory and GNG effect, with the approach and avoidance conditions combined (left column, the same as in Figure 12), when the consequence was approach (middle column), and when the consequence was avoidance (right column). Overall, the results remained largely the same for approach and avoidance consequences separately.

Figure 14 shows the correlations between AAT memory and AAT effect, with the go and no-go conditions combined (left column, the same as in Figure 12), when the response was go (middle column), and when the response was no-go (right column). We note one discrepancy from the previous results. That is, in Experiment 1 when the response was no-go (top row, right column in Figure 14), the correlation between AAT memory and AAT effect was not statistically significant. We note that these correlational results are exploratory in nature and need to be further examined in future work.

## 5.3 Summary and discussion

In the manuscript, we discussed why experimenter demands could not easily explain the observed effects on stimulus evaluation. As one reviewer argued, the correlations between effects of memory and effects on ratings observed here may argue in favor of experimenter demands.We agree that experimenter demand effects could potentially lead to a correlation between memory and effects on ratings. It is important to note, however, that this correlation has been observed in many other studies, where demand compliance is unlikely the only explanation. For instance, a similar correlation between memory and the go/no-go training effect on food choices has been observed in previous work (Chen & Veling, 2022). The effect of go/no-go training on food choices was less likely to be explained by demand because (1) the choices were consequential, such that participants' choices determined what they would receive and eat by the end of the experiment; (2) the choices probing the training effect were randomly embedded within other 'check' choices, and in these other 'check' choices subjects clearly showed a preference for snacks that they liked more, suggesting that they considered the value of the snacks when they made choices; and (3) the effect of go/no-go training became stronger when participants made choices more quickly, within 1.5 seconds (Chen et al., 2019, 2021; Wu et al., 2023), which does not fit with the typical time frame of demand-related processes. The effect of approach/avoidance training on real food choices has similarly been demonstrated with the same choice task (Veling et al., 2021). Furthermore, there is also some evidence that contingency memory moderates the effect of approach/avoidance training on implicit evaluation (measured with the evaluative priming task), which is arguably less susceptible to experimenter demand (Van Dessel et al., 2016). Experimenter demand is therefore not the only explanation for the observed correlation. Other plausible explanations also exist. For instance, such a correlation may arise, because participants who more strongly learn the contingencies in the task are also better able to (1) report these contingencies and (2) make inferences on the basis of these contingencies that produce the effect on stimulus evaluation (which is the crucial process underlying GNG and AAT effects according to inferential theories; Van Dessel et al., 2018). Again, such inferential processes do not mean that the effects we observed are driven by demand, as we have argued in the main text.

# 6 Ratings after the memory tasks

For exploratory purposes, participants received the same rating task again after the two memory tasks. Here we analyzed these rating data, using the same data analysis approach as in the manuscript. More concretely, we computed the change in rating from before the training to after the memory tasks, and submitted these change scores to a 2 (response, go vs. no-go) by 2 (consequence, approach vs. avoidance) repeated-measures ANOVA and a series of pairwise comparisons.

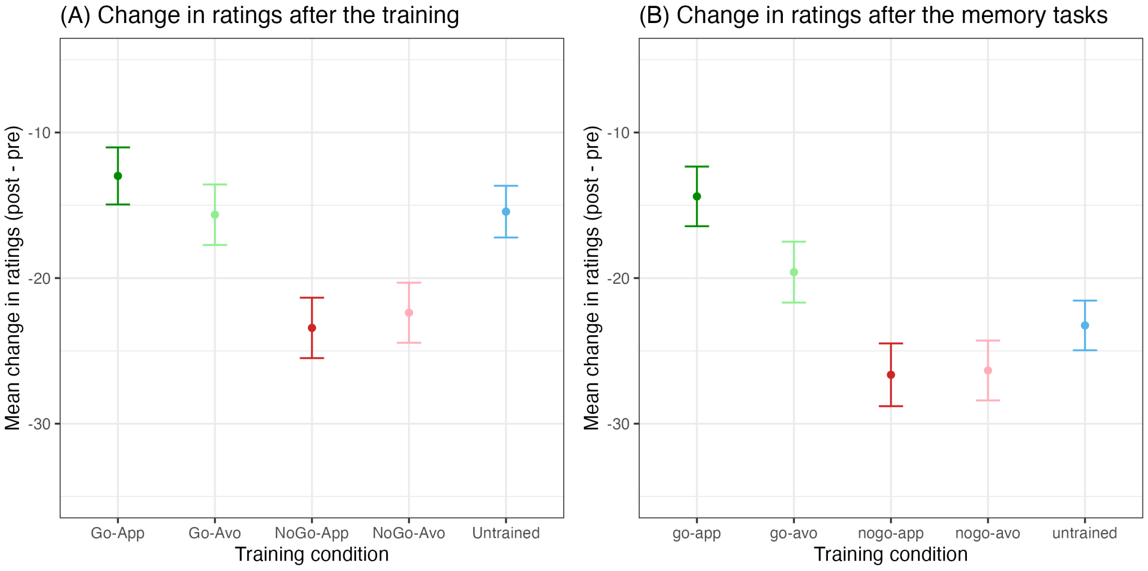


Figure 15: Changes in rating from before the training to immediately after the training (A) and after the memory tasks (B) in Experiment 1. The error bars stand for within-subject 95% confidence intervals. App = Approach, Avo = Avoidance.

Similar to the ratings immediately after the training, after the memory tasks, the ANOVA still revealed a main effect of go vs. no-go response, $F(1, 146) = 63.31$, $p < .001$, $ges = 0.059$, $BF_{10} = 1.434 \times 10^{10}$. The main effect of approach vs. avoidance consequence was statistically significant, $F(1, 146) = 5.04$, $p = .026$, $ges = 0.004$, but the Bayes factor was inconclusive, $BF_{10} = 1.138$. Lastly, different from the ANOVA on ratings immediately following training, the interaction effect was now also statistically reliable, $F(1, 146) = 9.39$, $p = .005$, $ges = 0.005$, $BF_{10} = 12.93$ (Figure 15).

Pairwise comparisons (Table 10) showed that overall no-go items were still evaluated less positively than both go and untrained items. However, the effect size for the difference between no-go and untrained items appeared to be smaller after the memory tasks than immediately after the training. Furthermore, we now also observed a go valuation effect, in that go items were evaluated more positively than untrained items. Overall, there was no effect of approach/avoidance consequence on evaluation. The comparisons between the four trained conditions against the untrained condition separately only revealed strong evidence for go-approach items to be more positive than untrained items, while for the remaining three comparisons the evidence was only anecdotal. The overall pattern of the results thus remained the same after the memory tasks in Experiment 1 (i.e., an overall GNG effect, but no AAT effect). However, measuring contingency memory appeared to reduce the no-go devaluation effect, and might have created a go valuation effect.

For Experiment 2, the results on the changes in rating after the memory tasks remained largely the same (Figure 16). After the memory tasks, there was still a main effect of approach/avoidance consequence, $F(1, 157) = 140.30$, $p < .001$, $ges = 0.112$, $BF_{10} = 2.895 \times 10^{20}$, no main effect of go/no-go response, $F(1, 157) = 2.64$, $p = .106$, $ges = 0.001$,

Table 10: Pairwise comparisons on changes in ratings (after the memory tasks) in Experiment 1.

| Comparison | diff | lowerCI | upperCI | df | t | p | BF | dz | gav |
|---|---|---|---|---|---|---|---|---|---|
| *Comparisons among the trained conditions* | | | | | | | | | |
| Go-App vs. NoGo-App | 12.25 | 9.23 | 15.27 | 146 | 8.02 | <.001 | $2.22 \times 10^{10}$ | 0.661 | 0.647 |
| Go-Avo vs. NoGo-Avo | 6.75 | 3.87 | 9.64 | 146 | 4.63 | <.001 | $1.70 \times 10^{3}$ | 0.382 | 0.351 |
| Go-App vs. Go-Avo | 5.20 | 2.54 | 7.87 | 146 | 3.86 | .002 | 96.23 | 0.318 | 0.292 |
| NoGo-App vs. NoGo-Avo | -0.30 | -3.21 | 2.62 | 146 | -0.20 | 1.000 | $9.37 \times 10^{-2}$ | 0.017 | 0.015 |
| *Comparisons against the untrained condition* | | | | | | | | | |
| Go-App vs. Untrained | 8.86 | 6.21 | 11.51 | 146 | 6.61 | <.001 | $1.34 \times 10^{7}$ | 0.545 | 0.536 |
| Go-Avo vs. Untrained | 3.66 | 0.88 | 6.44 | 146 | 2.60 | .143 | 2.36 | 0.215 | 0.212 |
| NoGo-App vs. Untrained | -3.39 | -5.96 | -0.82 | 146 | -2.60 | .142 | 2.38 | 0.215 | 0.185 |
| NoGo-Avo vs. Untrained | -3.09 | -5.65 | -0.53 | 146 | -2.39 | .255 | 1.43 | 0.197 | 0.173 |
| *Comparisons for the main effects of Go/NoGo* | | | | | | | | | |
| Go vs. NoGo | 9.50 | 7.14 | 11.86 | 146 | 7.96 | <.001 | $1.58 \times 10^{10}$ | 0.656 | 0.557 |
| Go vs. Untrained | 6.26 | 3.89 | 8.63 | 146 | 5.23 | <.001 | $2.05 \times 10^{4}$ | 0.431 | 0.394 |
| Untrained vs. NoGo | 3.24 | 1.13 | 5.36 | 146 | 3.03 | .040 | 7.32 | 0.250 | 0.190 |
| *Comparisons for the main effects of Approach/Avoidance* | | | | | | | | | |
| App vs. Avo | 2.45 | 0.29 | 4.61 | 146 | 2.25 | .368 | 1.05 | 0.185 | 0.146 |
| App vs. Untrained | 2.74 | 0.60 | 4.87 | 146 | 2.54 | .171 | 2.02 | 0.209 | 0.168 |
| Untrained vs. Avo | -0.28 | -2.53 | 1.97 | 146 | -0.25 | 1.000 | $9.47 \times 10^{-2}$ | 0.021 | 0.017 |

*Note.* P values were corrected for multiple comparisons using the Bonferroni method.

$BF_{10} = 0.305$, and also no interaction effect, $F(1, 157) = 1.52$, $p = .220$, $ges =< .001$, $BF_{10} = 0.268$.

Pairwise comparisons (Table 11) similarly showed that participants evaluated approached items more positively than untrained items, and avoided items less positively than untrained items. When the consequence was matched, there was no effect of go vs. no-go responses. The evaluation of no-go items also did not differ from that of go and untrained items. Note that the observed difference between go and untrained items was mostly caused by go-approach items being more positive than go-avoidance items. Overall, the results on ratings were thus highly stable from immediately after the training to after the memory tasks.

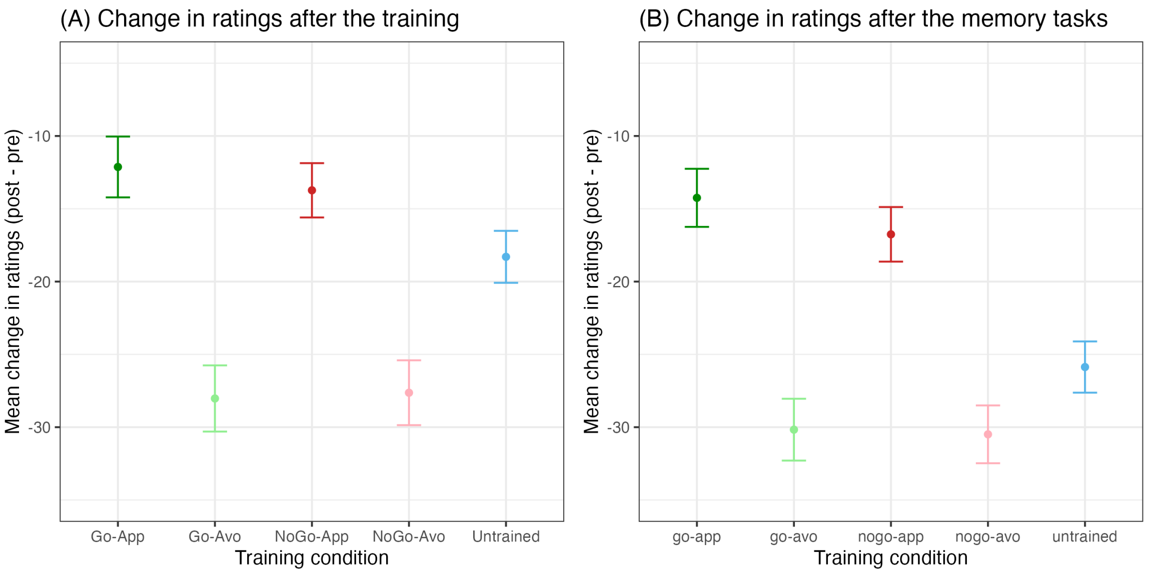


Figure 16: Changes in rating from before the training to immediately after the training (A) and after the memory tasks (B) in Experiment 2. The error bars stand for within-subject 95% confidence intervals. App = Approach, Avo = Avoidance.

# References


Diedenhofen, B., & Musch, J. (2015). Cocor: A Comprehensive Solution for the Statistical Comparison of Correlations. *PLOS ONE*, 12.

Van Dessel, P., De Houwer, J., & Gast, A. (2016). Approach–Avoidance Training Effects Are Moderated by Awareness of Stimulus–Action Contingencies. *Personality and Social Psychology Bulletin*, *42*(1), 81–93. https://doi.org/10.1177/0146167215615335

Van Dessel, P., Hughes, S., & De Houwer, J. (2018). How Do Actions Influence Attitudes? An Inferential Account of the Impact of Action Performance on Stimulus Evaluation. *Personality and Social Psychology Review*, 108886831879573. https://doi.org/10.1177/1088868318795730

Chen, Z., Holland, R. W., Quandt, J., Dijksterhuis, A., & Veling, H. (2019). When mere action versus inaction leads to robust preference change. *Journal of Personality and Social Psychology*, *117*(4), 721–740. https://doi.org/10.1037/pspa0000158

Chen, Z., Holland, R., Quandt, J., Dijksterhuis, A., & Veling, H. (2021). How Preference Change Induced by Mere Action Versus Inaction Persists Over Time. *Judgment and Decision Making*, *16*(1), 201–237. https://doi.org/10.31219/osf.io/b495y

Veling, H., Verpaalen, I. A., Liu, H., Mosannenzadeh, F., Becker, D., & Holland, R. W. (2021). How can food choice best be trained? Approach-avoidance versus go/no-go training. *Appetite*, *163*, 105226. https://doi.org/10.1016/j.appet.2021.105226

Chen, Z., & Veling, H. (2022). Toward a better understanding of durable behavior change by food Go/NoGo training. *Current Opinion in Behavioral Sciences*, *48*, 101212. https://doi.org/10.1016/j.cobeha.2022.101212

Houben, K. (2023). How does Go/No-Go training lead to food devaluation? Separating the effects of motor inhibition and response valence. *Cognition and Emotion*, 1–14. https://doi.org/10.1080/02699931.2023.2208339

Table 11: Pairwise comparisons on changes in ratings (after the memory tasks) in Experiment 2.

| Comparison | diff | lowerCI | upperCI | df | t | pt | BF | dz | gav |
|---|---|---|---|---|---|---|---|---|---|
| *Comparisons among the trained conditions* | | | | | | | | | |
| Go-App vs. NoGo-App | 2.51 | 0.19 | 4.82 | 157 | 2.14 | 0.478 | 0.809 | 0.170 | 0.131 |
| Go-Avo vs. NoGo-Avo | 0.32 | -2.26 | 2.90 | 157 | 0.25 | 1.000 | $9.13 \times 10^{-2}$ | 0.019 | 0.014 |
| Go-App vs. Go-Avo | 15.92 | 12.76 | 19.09 | 157 | 9.94 | 0.000 | $2.23 \times 10^{15}$ | 0.791 | 0.773 |
| NoGo-App vs. NoGo-Avo | 13.74 | 10.85 | 16.63 | 157 | 9.38 | 0.000 | $8.05 \times 10^{13}$ | 0.747 | 0.644 |
| *Comparisons against the untrained condition* | | | | | | | | | |
| Go-App vs. Untrained | 11.62 | 9.14 | 14.10 | 157 | 9.25 | 0.000 | $3.59 \times 10^{13}$ | 0.736 | 0.584 |
| Go-Avo vs. Untrained | -4.30 | -7.14 | -1.46 | 157 | -2.99 | 0.045 | 6.42 | 0.238 | 0.201 |
| NoGo-App vs. Untrained | 9.12 | 6.46 | 11.77 | 157 | 6.79 | 0.000 | $3.89 \times 10^{7}$ | 0.540 | 0.456 |
| NoGo-Avo vs. Untrained | -4.62 | -7.14 | -2.10 | 157 | -3.62 | 0.006 | 42.68 | 0.288 | 0.210 |
| *Comparisons for the main effects of Go/NoGo* | | | | | | | | | |
| Go vs. NoGo | 1.41 | -0.30 | 3.13 | 157 | 1.63 | 1.000 | 0.322 | 0.129 | 0.076 |
| Go vs. Untrained | 3.66 | 1.51 | 5.81 | 157 | 3.37 | 0.013 | 19.39 | 0.268 | 0.189 |
| Untrained vs. NoGo | -2.25 | -4.39 | -0.10 | 157 | -2.07 | 0.565 | 0.704 | 0.164 | 0.112 |
| *Comparisons for the main effects of Approach/Avoidance* | | | | | | | | | |
| App vs. Avo | 14.83 | 12.36 | 17.30 | 157 | 11.84 | 0.000 | $2.63 \times 10^{20}$ | 0.942 | 0.762 |
| App vs. Untrained | 10.37 | 8.08 | 12.66 | 157 | 8.93 | 0.000 | $5.71 \times 10^{12}$ | 0.711 | 0.540 |
| Untrained vs. Avo | 4.46 | 2.11 | 6.82 | 157 | 3.74 | 0.004 | 64.80 | 0.298 | 0.213 |

*Note.* P values were corrected for multiple comparisons using the Bonferroni method.

Liu, H., Holland, R., & Veling, H. (2023). When not responding to food changes food value: The role of timing. *Appetite*, 106583. https://doi.org/10.1016/j.appet.2023.106583

Wu, Q., Xia, H., Shields, G. S., Nie, H., Li, J., Chen, H., & Yang, Y. (2023). Neural correlates underlying preference changes induced by food Go/No-Go training. *Appetite*, *186*, 106578. https://doi.org/10.1016/j.appet.2023.106578
